# Alendronate repositioning as potential anti-parasitic agent targeting *Trichinella spiralis* inorganic pyrophosphatase, in vitro supported molecular docking and molecular dynamics simulation study

**DOI:** 10.1186/s13065-025-01468-4

**Published:** 2025-05-06

**Authors:** Marmar A. Hanafy, Doaa A. Nassar, Fatima M. Zahran, Magdy M. D. Mohammed

**Affiliations:** 1 Department of Parasitology, Faculty of Medicine, Ain Shams University, Cairo, Egypt; 2 Department of Pharmacognosy, Pharmaceutical and Drug Industries Research Institute, National Research Centre, Dokki, Cairo, 12622 Egypt

**Keywords:** Alendronate, Albendazole, *Trichinella spiralis*, Scanning electron microscopy, Molecular docking, Molecular dynamics simulation

## Abstract

Trichinellosis represents great public health and economic problems worldwide. Moreover, the development of parasitic resistance against conventional anthelminthic treatment led to the urgent search for new therapeutic strategies, including drug repurposing. Bisphosphonates have been used to inhibit the growth of many parasites and have also emerged as promising candidates for the treatment of cryptosporidiosis and amoebic liver abscess. Alendronate is a second-generation bisphosphonate that is widely used for the treatment and prevention of osteoporosis. Till date, there is not enough data on the effect of this drug on *Trichinella spiralis* and it is unknown whether the regular use of this drug in osteoporotic patients may alter the course of the infection. ALN showed a significant lethal effect on both adult worms and juveniles, with severe tegumental damage in the form of fissures in the cuticle, widening of the hypodermal gland, and flattening of the cuticular annulation, ending with the appearance of multiple vesicles and large cauliflower masses. Molecular docking outcomes unveiled the potential inhibition of ALN against *T. spiralis* surface proteins (*i.e., Ts*-SP, *Ts*-PPase, *Ts*-MAPRC2, *Ts*-TS, *Ts*-MIF, etc.), with promising results confirmed its ability to defeat *T. spiralis* via targeting its surface proteins. Moreover, molecular dynamics simulation, through the analysis of RMSD, RMSF, RG, SASA and cluster analysis, proved the prolonged effective inhibition of ALN on *T. spiralis* inorganic pyrophosphatase, as an essential surface protein required for molting and developmental process of intestinal larval stages. Thus, ALN might be a valuable drug candidate for the treatment of trichinellosis and warrant further investigation in animal models of disease.

**Supplementary Information:**

The online version contains supplementary material available at 10.1186/s13065-025-01468-4.

## Introduction

Trichinellosis is a foodborne zoonotic parasitic disease caused by nematode worms of the genus *Trichinella* [1], which infects a wide range of hosts including; birds, domestic and wild mammals, reptiles and humans [2]. Humans mostly acquire the infection by *Trichinella spiralis* through the consumption of raw or undercooked pork meat that contains *T. spiralis* infective muscle larvae (ML) [3]. Trichinellosis represents a great public health concern for humans, in addition to the financial costs associated with controlling the disease in sensitive animals [4].

Currently, benzimidazole derivatives albendazole (ABZ) and mebendazole (MBZ) are the two primary anthelmintic drugs used to treat trichinellosis [5]. However, even with their high effectiveness against adult worms (AW) [6], these drugs have some limitations, that are summarized in their low water solubility which affects their bioavailability and leading to poor efficacy against encapsulated and newborn larvae [7]. Furthermore, most benzimidazoles are contraindicated for pregnant women and children below two years of age [8], in addition to, severe side effects that have been reported including acute liver injury, anemia and leukopenia [9]. Given the limitations of the currently available drugs used for the treatment of trichinellosis, besides, there is an increasing concern about the parasite resistance development against ABZ [10], which calls the need for the development of more effective, safe, and better-tolerated new anthelmintic agents against *Trichinella* [11].

Drug repurposing has recently emerged as a novel strategy for bringing new applications for pre-existing commercially approved and/or rejected drugs, in such a way, to treat diseases apart from their initial therapeutic indications [12]. Alendronate sodium is the sodium salt of alendronate (ALN), a second-generation bisphosphonate, it is the synthetic analog of pyrophosphate with bone anti-resorption activity, and mainly approved as an oral available therapeutic agent for the treatment of primary or corticosteroid-induced osteoporosis at low cost [13], as well as for the prevention of osteoporosis in postmenopausal women [14]. A wide safety margin of ALN usage ranging from 5 mg daily for osteoporosis prevention and 10 mg daily/70 mg weekly for treatment. Moreover, adverse events are generally transient and primarily associated with the upper GI tract, most commonly including abdominal pain, nausea, and dyspepsia [14–16]. ALN is generally well tolerated when taken at recommended doses and with specific instructions designed to minimize the risk of upper GI side effects. Moreover, serious side effects, such as atypical femur fractures, are rare and are mainly observed with long-term use exceeding five years [17], a condition not relevant to the treatment of trichinellosis.

Additionally, derivatives of bisphosphonates, *i.e.,* ALN, have been proven to show antibacterial [18], herbicidal, anticancer [19] and antiparasitic properties [20]. In addition, they have been reported to inhibit the growth of many parasites including; *Leishmania species*, *Plasmodium falciparum, Schistosoma mansoni*, *Toxoplasma gondii* and *Trypanosoma brucei*, [20–23]. Furthermore, ALN may offer additional advantages in the treatment of trichinellosis beyond the standard anthelmintic therapies, these potential benefits include its anti-inflammatory properties [24], which could help alleviate muscular complications associated with trichinellosis, as well as its inhibitory effects on angiogenesis, which may impact muscle larvae and the formation of nurse cells [25]. Moreover, they have also emerged as promising candidates for the treatment of amoebic liver abscess and cryptosporidiosis [26, 27]. Surprisingly, repurposing of ALN to target *T. spiralis* surface proteins as a possible inhibitory drug has not tried till date.

*T. spiralis* ES surface proteins are located at the interface between the parasite and the host helping to modify the surrounding environment, through the modulation of the host immune response or even host cell gene expression, which in turn led to ensure parasite invasion, development and survival, and represent the defense line in front of the host’s immune system, therefore, they are considered as the main target antigens that bring the immune responses, moreover, the analysis and characterization of *T. spiralis* surface proteins could provide useful information to elucidate the host-parasite interaction, identify the early diagnostic antigens, and identify the targets for vaccines [28].

For the time being, comprehensive computational pipelines [29], including molecular docking to evaluate the potential interactions between the receptor and ligands through their binding affinities and molecular interactions [30, 31], moreover, molecular dynamics simulations give detailed information regarding the stability, folding and conformational changes of the resulted complexes, through the use of advanced analytical tools to unveil the detailed protein–ligand dynamics at their atomic level [32, 33]. Todays, computational software and web servers are used for analyzing, capturing, and integrating medical data from diverse sources also called computational pharmacology [34–37]. Considering the above mentioned, the present study was attempted to investigate the in vitro effect of ALN on *T. spiralis* AW and ML, in comparison with ABZ, and in order to clarify their probable modes of action, an in silico molecular docking study targeting *T. spiralis* surface proteins (*i.e., Ts*-SP, *Ts*-PPase, *Ts*-MAPRC2, *Ts*-TS, *Ts*-MIF, etc.) was performed, additionally, the molecular dynamics behavior of the resulted complexes was detailed investigated via the analysis of RMSD, RMSF, RG, SASA and cluster analysis of their H-binding interactions.

## Methods

### In vitro study

This study was carried out at Theodor Bilharz Research Institute (TBRI), Giza, Egypt, during August 2023 – April 2024. Different concentrations of ALN were tested against *T. spiralis* AW and ML and the percent of the mortality rate was determined by using light microscopy, then the morphological changes of AW and ML were detected by SEM in comparison to ABZ.

### Parasite

*T. spiralis* adult worms and muscle larvae were isolated from the laboratory-bred infected Swiss albino mice, which were purchased from Theodor Bilharz Research Institute (TBRI), Giza, Egypt. Mice were orally infected by 200–300 *T. spiralis* larvae, then the infected mice were housed under standard conditions including 45:50 relative humidity, with 12 h light/dark cycle and at temperature of 22 ± 2 °C. Mice were fed on a standard pelleted diet ad libitum, with free access to water throughout the experimental period [38]. Generally, the AW were isolated five days post-infection (dpi), while ML were collected 35 dpi [7].

Briefly, isolation of the AW was performed as follow; ten infected mice were sacrificed by cervical dislocation under light anesthesia by isoflurane inhalation (Sigma-Aldrich, USA), Cat#792,632, then the intestines of the infected mice were removed and washed up, the cleaned intestines were then longitudinally opened with scissors, cut into 2 cm sections, and then submersion in 250 mL phosphate-buffered saline (PBS) and left for 3 h at 37 °C [39]. The ML were isolated through the dissection of each mouse to get the muscles, which were digested in an acid pepsin solution (200 mL of distilled water with 1% conc. HCl and 1% pepsin). The resulted mixture was stirred via electric stirrer for 2 h at 37 °C, followed by filtration through a 200-mesh/inch screen to collect the released larvae. The collected larvae were washed up in tap water three times and then were allowed to sediment in a conical flask containing 150 mL of tap water for half an hour. Finally, the supernatant fluid was discarded, and the sedimented larvae were collected and counted using a McMaster counting chamber [40].

### Drugs

ALN was purchased as Bonapex tablets (70 mg) from APEX Pharma. The tablets were ground and the obtained powder was suspended in distilled water to prepare a stock solution of 500 μM. Then gradient concentrations of serial dilutions: 49.6, 24.8, 12.4, 6.2 and 3.1 μg/mL were prepared [18]. ABZ tablets (400 mg) (Pharco Pharmaceuticals, Egypt) were crushed and dissolved in 1% dimethyl sulphoxide (DMSO) to prepare a stock solution of 400 μg/mL. The stock solution was diluted in DMSO to prepare working solutions of 50 and 25 μg/mL [41].

### Experimental design

The freshly recovered AW and/or ML were separately incubated in a 24-well culture plate (Corning, New York, NY, USA), containing a sterile RPMI-1640 medium supplemented with 5% fetal calf serum (FCS), 200 μg/mL streptomycin and 200 IU/mL penicillin. The prepared parasite suspension contained 15 AW and/or 30 ML in 2 mL of the prepared culture medium. The plate was sealed and incubated at 37 °C and 5% carbon dioxide for 96 h [42].

### Study grouping

The incubated AW and ML were grouped into: *Group I*: included the AW cultured in an incubation medium containing ALN solution at different concentrations: 3.1, 6.2, 12.4, 24.8 and 49.6 μg/mL. *Group II*: included ML cultured in an incubation medium containing ALN solution at different concentrations: 3.1, 6.2, 12.4, 24.8 and 49.6 μg/mL. *Group III*: included the AW cultured in an incubation medium containing ABZ at concentrations: 25 and 50 μg/mL. *Group IV*: included ML cultured in an incubation medium containing ALB at concentrations: 25 and 50 μg/mL.

Additionally, adult/larva negative control group: containing AW or ML cultured in drug-free incubation medium and adult/larva DMSO control group: containing AW or ML cultured in incubation medium containing 1% DMSO. The experiment was performed in triplicate for each drug concentration.

### Viability evaluation

The viability of the incubated worms, at various drug concentrations, was evaluated using light microscopy, in comparison with the control groups. Worms were checked and counted out at 1, 3, 6, 12, 24, 48, 72 and 96 h, to investigate their motility and figure out the lives and dead ones, thus, worms of no motility for several minutes were detected to be dead [22]. Results were expressed as means ± SD, and the mortality rate was then estimated and recorded according to the following equation formula [43]:

Mortality rate (%) = (Number of dead AW or ML/Total number of AW or ML in each well) × 100.

### Scanning electron microscopy

To demonstrate the destructive effect of ALN and ABZ on the ultrastructure of *T. spiralis* AW and ML, the concentrations of ALN and ABZ that induced the highest mortality rate at 48 h of incubation were used. Worms from each group were directly pipetted and immediately fixed in a fresh fixation solution of 2.5% glutaraldehyde buffered with 0.1 M sodium cacodylate at pH 7.2 and left overnight at 4 °C. Fixed *Trichinella* stages were then washed in 0.1 M sodium cacodylate buffer at pH 7.2 for 5 min, followed by 1 h fixation in 2% osmium tetroxide, then washed in distilled water. The specimens were dehydrated in series of ascending ethanol concentrations, mounted on double-sided carbon-coated adhesive tape, and examined using a scanning electron microscope (Jeol GSM-IT200, Japan) available at the Electronic Microscope Unit, Faculty of Science, Alexandria University [44].

### Statistical analysis

IBM SPSS software package version 20.0. (Armonk, NY: IBM Corp) was used to analyze the resulted data, and it were expressed as mean and standard deviation. One way ANOVA test was used to compare between the different studied groups and followed by Post Hoc test (Tukey) for pairwise comparison. The significance of the obtained results was judged at the 5% level.

### In silico study

#### Molecular docking

PyRx version 0.8 software, a built on open-source tools and libraries, including AutoDock Vina, Open Babel, etc., with Lamarckian genetic algorithm (LGA) as scoring function, was used for performing molecular docking [45]. Albendazole and alendronate 3D structures in.sdf format were retrieved from the PubChem (https://pubchem.ncbi.nlm.nih.gov). Following up, ABZ and ALN were converted into.pdb format using Open Babel and submitted to PyRx for energy minimization to ensure their optimal suitability [46]. Post-minimization, the ligands were converted to.pdbqt format. Grid box parameters covering the chosen pocket (Table 1) were served as the focal point for docking calculations and exhaustiveness were kept at 100. The 3D binding interactions of protein–ligand complexes were visualized using PyMol package, in addition, the 2D interactions were visualized in LigPlot^+^ V 2.2 [47].

**Table 1:** Molecular interactions of albendazole and alendronate at the active site of *T. spiralis* surface proteins

Receptor Proteins (IDs)	Albendazole			Alendronate			Grid box parameters Center (x y z)/ Size (x y z)
	∆G (kcal/mol)	No. HB	Receptor AA Involved (Distance Å)	∆G (kcal/mol)	No. HB	Receptor AA Involved (Distance Å)	
*Ts*-MIF (1hfo)	−6.3	0	Hyd: Pro1, Ile2, Lys32, Tyr36, Ile64, Val106, Gly107, Trp108, Phe113	−4.7	3	HB: Pro1(3.32), Tyr36(2.74 & 2.66), Gly107(2.93) Hyd: Ile2, Val37, Ala38, Ile64, Val106, Trp108, Phe113	33.32 9.89 18.56/ 16.22 21.35 18.85
*Ts*-calreticulin (8xvf)	−6.2	2	HB: Asp63(2.88), Gln350(3.02) Hyd: His57, Phe61, Ala62, Asp66, Lys349, Ala353, Leu354	−5.4	8	HB: Ser56(2.90), His57(2.88 & 2.91), Leu60(2.80), Asp66(2.53), Thr100(3.31), Lys102(3.22 & 3.24), Thr173(3.18), Asp332(3.16) Hyd: Phe61, Gly68, Ile330, Asn333, Leu351, Leu354	25.01 8.49 43.05/ 21.79 19.84 20.30
*Ts-*thymidylate synthase (5by6)	−6.5	1	HB: Leu215(3.13) Hyd: Arg72, Phe74, Glu81, Ile102, Trp103, Gly216, Phe219	−4.8	3	HB: Glu81(2.89 & 2.92), Leu215(2.87), Asn220(2.80) Hyd: Ile102, Trp103, Gly216, Phe219	1.72 15.74 14.56/ 25.41 21.99 23.67
*Ts*-PPase (6C45)	−6.8	3	HB: Glu141(3.34), Asp230(2.85 & 3.31), Tyr275(3.16) Hyd: Lys139, Ile163, Tyr172, Tyr176, Asp198, Cys205, Asp235, Lys237, Leu272	−5.5	9	HB: Glu131(3.22), Lys139(3.29), Glu141(2.70), Tyr176(2.70), Asp198(2.93 & 3.09), Asn200(2.77), Asp203(3.03 & 3.07), Asp235(2.75, 2.79 & 3.08), Lys237(3.00)	21.61 63.13 38.50/ 20.56 25.00 25.00
*Ts*-CF1 (1m6d)	−5.2	1	HB: Gln167(3.06) Hyd: Gly168, Cys170, Gly171, Cys173, Met288, His309, Trp335, Trp339	−4.3	5	HB: Gln167(2.85), Gly168(3.04 & 3.31), Cys170(3.28), His309(3.30), Trp335(2.95) Hyd: Gly171, Cys173	6.07 12.42 9.43/ 14.16 19.10 16.54
*Ts*-MAPRC2 (4xy8)	−4.6	1	HB: Arg150(2.98 & 3.17) Hyd: Arg109, Asp112, Gln140, Gly142, Gly145, Leu146	−4.3	5	HB: Asp112(2.94 & 3.02), Gly145(3.01 & 3.24), Leu146(2.99), Ala148(2.89), Arg150(2.84 & 3.01) Hyd: Gln140, Met163	46.56 26.83 66.13/ 17.97 19.21 18.77
*Ts*-Serine proteinase (5cdz)	−4.0	1	HB: Lys245(2.87) Hyd: Glu177, Pro179, Glu182, Thr183, Phe185, Leu246	−3.6	2	HB: Glu177(2.76 & 2.78), Tyr219(2.76 & 3.04) Hyd: Tyr180, Arg209, Ser210, Phe222, Lys273	−14.97 35.63 −12.57/ 18.54 19.30 16.21

### Receptors preparation

#### The X-ray crystallographic structures

The X-ray crystallographic structures of *Ts*-MIF, *Ts*-calreticulin, and *Ts-*thymidylate synthase (PDB IDs: 1hfo, 8xvf, and 5by6, respectively) were downloaded from RCSB, then water molecules and other hetero atoms were removed, then after, the polar hydrogen atoms were added to the 3D structures in a process of protein refinement using PyMol, followed by, both of protein and ligands were underwent minimization and conversion to the.pdbqt format using PyRx.

### Prediction of the 3D structures of *Ts*-surface proteins

Noteworthy, the lack of the available 3D crystal structures of *T. spiralis* surface proteins, prompted our group to construct 3D structures based on their available amino acid sequences, using template-based protein modeling (TBM), which encompasses, the homologous identification, then the target to template alignment, followed by building up the 3D-structure, and finally, the refinement and validation. Briefly, the amino acid sequences of *T. spiralis* cathepsin F (*Ts*-CF1), *Ts*-serine proteinases, inorganic-pyrophosphatase (*Ts-*PPase) and *T. spiralis* membrane-associated progesterone receptor (*Ts*-MAPRC2) were retrieved from the NCBI GenBank database on the FASTA format (GenBank: XM_003378197.1, XP_003376000.1, XP_003371891.1 and XP_003375934.1 respectively). Followed by, a homology modeling approach to build up the 3D structures using protein fold recognition server (PHYRE2, ic.ac.uk). The generated 3D-structures were energy minimized using YASARA force field (YASARA Energy Minimization Server) for improvement, after that, the generated models were assessed using ERRAT (protein structures verification using crystallography and modeled proteins), general parameters for stereochemical quality by PROCHECK (protein residues with possible conformations of the ψ and φ angels), and VERIFY-3D (characterizes the solidarity of 3D-model with its 1D-amino acid sequence) of the SAVES server. Additionally, the energy-minimized models were examined by the Ramachandran plots.

### Prediction of the active binding site

Binding site determination is a crucial step to figure out the key amino acid residues, at which the ligand binds to exert receptor inhibition, an easy way with a co-crystallized ligand, however, the active binding sites of all the targeted receptors were determined using site map and site finder modules.

### Molecular dynamics (MD) simulation

Understanding the long-lasted behavior of a protein–ligand complex requires high-throughput dynamic simulation study, in order to confirm docking findings, prove complex stability and to achieve the optimal configuration.

NAMD simulation software (Ver. 3.0.1, 2024. WSL-Ubuntu) and VMD package were used for MD simulation and system preparation respectively [48]. Input-generator plugin in web-based platform (CHARMM-GUI) was used to generate the ligand topology files. Solvated and ionized files (PSFs and PDBs) of the systems were generated by adding water molecules (box of 11 Å thickness) and 0.15 Mol/L of NaCl respectively [49–51]. Briefly, conjugate gradient approach was used for 20 ps at 300 K for energy minimization with constant pressure. Switching function, using values of 8.0 and 12.0 Å for the switchdist and cutoff respectively, was used for molecular interactions including electrostatic, long-range and van der Waals. The SHAKE algorithm was used to constrain bond [52] and the calculations of the long and short-range electrostatic forces were done using PME (particle mesh) method [53]. Hence, system was heated up for 600 ps with 1 fs time step via increasing the temperature from 0 to 300 K, linearly with 0.001 K, and Maxwell distribution was used to reassign particles velocities. Both of Langevin piston and Hoover thermostat were used to control pressure and temperature respectively, throughout the equilibration of 1 ns with time step of 2 fs and 1 ps frame record [54, 55]. Finally, statistical NVE ensemble was used for 100 ns production in triplicates to figure-out the MD trajectory files.

### Structural stability investigation using trajectory analysis

The resulted DCD trajectory files were used to study the structural changes, function and stability of the complexes, through the analyses of RMSD (receptor excluding hydrogens) using VMD plugin, moreover, RMSF (chain-C*α* residues), RG and SASA were calculated using TCL [56].

### Hydrogen bond analysis

The hydrogen bond analysis was carried out using H-bonds plugin in VMD. Considering all hydrogen bonds involved in the protein–ligand complex, especially those achieving the bond distance of 3 Å and angle of 20º, to acquire only strong and prolonged hydrogen bonds.

### Cluster analysis

The g_cluster tool in GROMACS was used to analyze the resulted MD trajectory with a cutoff radius of 2 Å [57]. Concisely, VEGAZZ software (http://www.vegazz.net/) was used to convert the DCD trajectory files (obtained from NAMD) into GROMACS readable XTC format, followed by a re-numbering step using a trjconv command. With an RMSD threshold of 0.11 nm the g_cluster tool was used to cluster all frames of each trajectory file. The representative structure, that showed the maximum number of frames, was extracted, and with trjconv command it was converted into pdb format, that requires for 2D interpretation using LigPlot^+^ software. The generated plots can be used to indicate the stability provided to the structure as a result of possible stable hydrogen bonds and hydrophobic contacts formed during *Ts*-PPase inhibition by ABZ and ALN.

## Results and discussion

Concisely, as outlined previously that ABZ, a primary anthelmintic drug, has high efficacy against *T. spiralis* adult worms (AW), but its performance is hampered by low solubility and physicochemical properties. In addition to some limitations including poor effectiveness against encapsulated and newborn larvae, besides its contraindication for pregnant women and children at certain age, along with, the severe side effects including acute liver injury, anemia and leukopenia.

That thing calls the need for discovering better-tolerated new effective and safe anthelmintic agents, which prompted our group to study the possibility for repurposing the anti-osteoporosis drug ALN, a 2nd generation of bisphosphonate, as a new antiparasitic agent, putting into account the distinguished antibacterial, herbicidal and antiparasitic activities of BPs. Interestingly, ALN revealed promising in vitro anti-*T. spiralis* AW and ML, with different modes of actions represented in its inhibition of *Ts-*PPase which is discussed extensively following.

## In vitro study

### Effect of ALN and ABZ on mortality rate of *T. spiralis* AW and ML

**Fig. 1:**
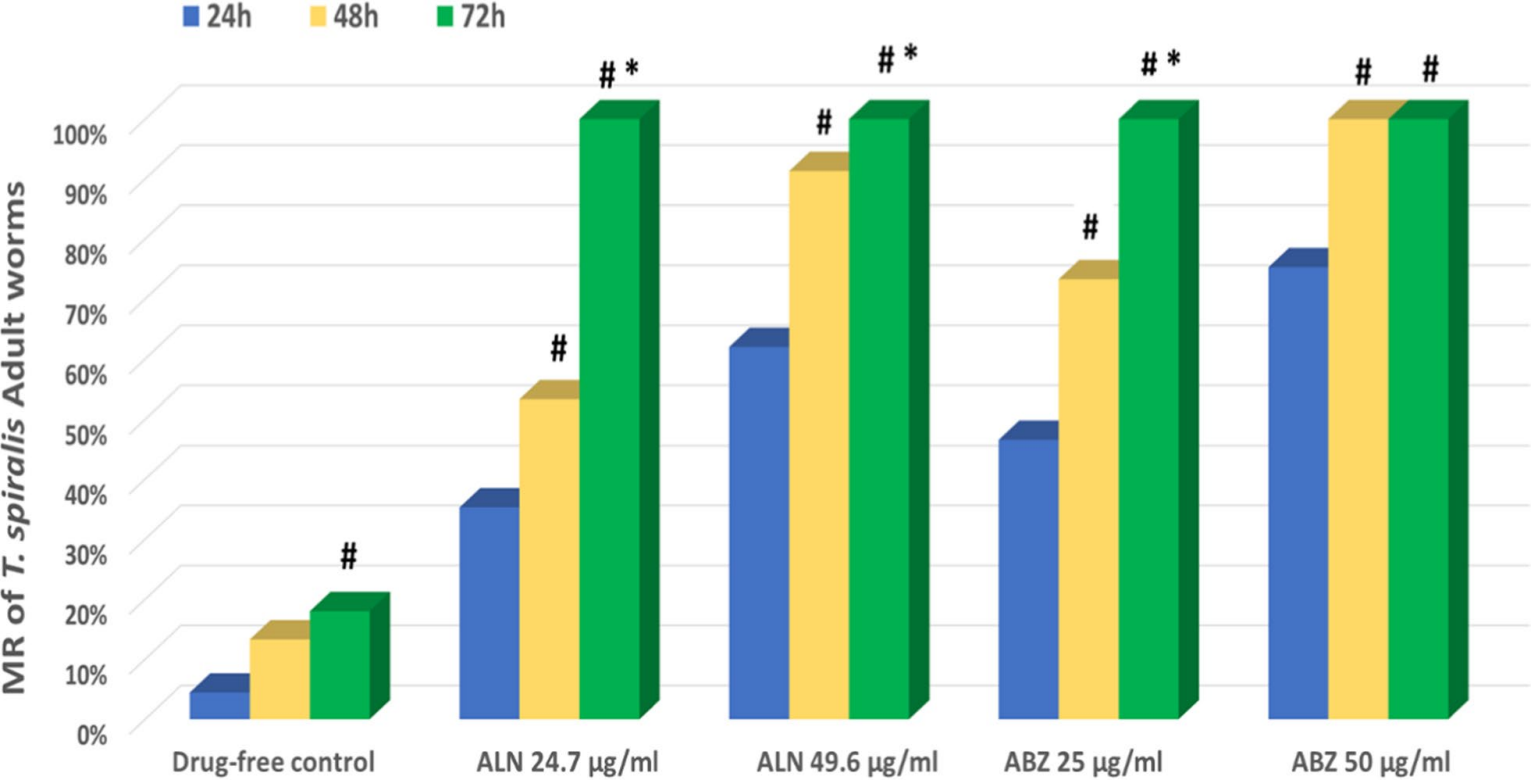
Comparison between the mortality rate (%) of *T. spiralis* AW in ALN (Group II) and ABZ (Group IV) treated groups in comparison to the drug-free control group at different times intervals. #: Significant versus the mean number of dead adult worms at 24 h. *: Significant versus the mean number of dead adult worms at 48 h and 72 h

**Fig. 2:**
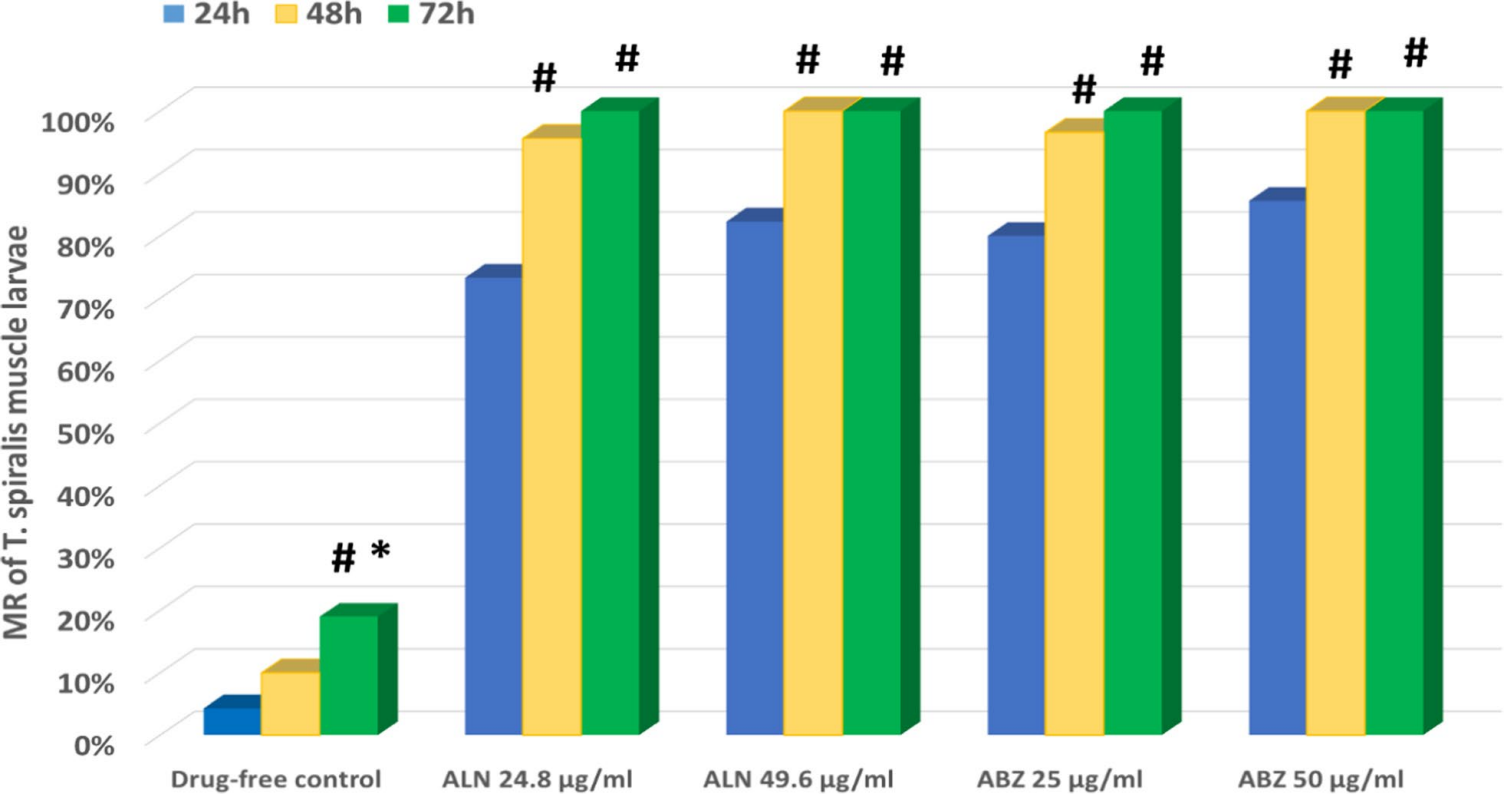
Comparison between the Mortality rate (%) of *T. spiralis* larvae in ALN (Group II) and ABZ (Group IV) treated groups in comparison to the drug-free control group at different times intervals. #: Significant versus the mean number of dead adult worms at 24 h. *: Significant versus the mean number of dead adult worms at 48 h and 72 h

Light microscope detection of *T. spiralis* AW and ML demonstrated 98% viability at the starting. To this end, over a time period of 96 h, significant differences were observed in the average mortality rates for AW and ML upon treatment with different concentrations of ALN and ABZ at different incubation intervals, in comparison to the drug-free adult and larval control groups (P < 0.001) (Tables 2 & 3). Concisely, the first 12 h of incubation revealed lethal effects of ALN on *T. spiralis* AW and ML at concentrations of 24.8 and 49.6 µg/mL (Figs. 1, 2). Furthermore, incubation of 24.8 µg/mL of ALN for 48 h increased the mortality rates to 53.3% and 95.6% for AW and ML, respectively, however, 49.6 µg/mL of ALN induced mortality with 91.3% for AW and 100% for ML, additionally, after 72 h of incubation with ALN, 100% death of AW and ML was recorded. On the other hand, 96 h of incubation with ALN at lower concentrations of 3.1, 6.9 and 12.4 µg/mL, produced AW mortality rates of 66.6%, 73.3% and 84.6%, respectively, and ML mortality rates of 63.3%, 75.6%, and 95.6%, respectively. These findings were in accordance with Ziniel et al. [22], who illustrated that, 4–6 days of incubation with bisphosphonates induced full death of adult *Schistosoma mansoni* worms. More reports by Martin et al. [58] and Branco Santos et al. [59] demonstrated growth suppressing effect of bisphosphonate-based molecules on *Trypanosoma brucei, T. cruzi, Leishmania donovani, Toxoplasma gondii*, *Plasmodium falciparum* and *Cryptosporidium spp*.

**Table 2:** Death numbers and mortality rates (%) of *T. spiralis* AW incubated with different concentrations of ALN and ABZ over a 96-h period

Adult			1 h	3 h	6 h	12 h	24 h	48 h	72 h	96 h
Drug-free Control		Mean ± SD	0^a^ ± 0	0^a^ ± 0	0^c^ ± 0	0^d^ ± 0	0.67^c^ ± 0.58	2^d^ ± 1	2.7^e^ ± 0.58	3^d^ ± 1
		MR	0%	0%	0%	0%	4.46%	13.3%	18%	20%
DMSO Control		Mean ± SD	0^a^ ± 0	0^a^ ± 0	0^c^ ± 0	0^d^ ± 0	1^c^ ± 1	2.3^d^ ± 0.58	3^e^ ± 1	3.7^d^ ± 0.58
		MR	0%	0%	0%	0%	6.66%	15.3%	20%	24.6%
ALN (µg/ml)	3.1	Mean ± SD	0^a^ ± 0	0^a^ ± 0	0^c^ ± 0	0^d^ ± 0	1^c^ ± 1	2.3^d^ ± 0.58	5.3^d^ ± 0.58	10^c^ ± 1
		MR	0%	0%	0%	0%	6.66%	15.3%	35.3%	66.6%
	6.2	Mean ± SD	0^a^ ± 0	0^a^ ± 0	0^c^ ± 0	0^d^ ± 0	1^c^ ± 1	3^d^ ± 1	8^c^ ± 1	11^bc^ ± 1
		MR	0%	0%	0%	0%	6.66%	20%	53.3%	73.3%
	12.4	Mean ± SD	0^a^ ± 0	0^a^ ± 0	0^c^ ± 0	1^ cd^ ± 1	2.7^c^ ± 0.58	7.3^c^ ± 0.58	10^b^ ± 1	12.7^b^ ± 0.58
		MR	0%	0%	0%	6.66%	18%	48.6%	66.6%	84.6%
	24.8	Mean ± SD	0^a^ ± 0	0^a^ ± 0	0^c^ ± 0	2.3^bc^ ± 0.58	5.3^b^ ± 0.58	8^c^ ± 1	15^a^ ± 0	15^a^ ± 0
		MR	0%	0%	0%	15.3%	35.3%	53.3%	100%	100%
	49.6	Mean ± SD	0^a^ ± 0	0^a^ ± 0	0^c^ ± 0	7^a^ ± 1	9.3^a^ ± 0.58	13.7^a^ ± 0.58	15^a^ ± 0	15^a^ ± 0
		MR	0%	0%	0%	46.6%	62%	91.3%	100%	100%
ABZ (µg/ml)	25	Mean ± SD	0^a^ ± 0	0^a^ ± 0	1.7^b^ ± 0.58	4^b^ ± 1	7^b^ ± 1	11^b^ ± 1	15^a^ ± 0	15^a^ ± 0
		MR	0%	0%	11.3%	26.6%	46.6%	73.3%	100%	100%
	50	Mean ± SD	0^a^ ± 0	0^a^ ± 0	3.7^a^ ± 0.58	8.7^a^ ± 0.58	11.3^a^ ± 0.58	15^a^ ± 0	15^a^ ± 0	15^a^ ± 0
		MR	0%	0%	24.6%	58%	75.3%	100%	100%	100%
p-value			–	–	< 0.001^**^	< 0.001^**^	< 0.001^**^	< 0.001^**^	< 0.001^**^	< 0.001^**^

*MR* mortality rate

Mean number of adults worms in each group is 15

Three replicas for each group. Data was expressed using Mean ± SD

F: F for One way ANOVA test, pairwise comparison between each 2 groups were done using Post Hoc Test (Tukey)

Different small letters^(a−e)^ indicate significant difference at (p ≤ 0.05) between study groups

Similar small letters^(a−e)^ indicate non-significant difference between study groups

p-value > 0.05 is non-significant, *p-value ≤ 0.05 is statistically significant, ** p-value < 0.001 is highly significant

**Table 3:** Death numbers and Mortality rates (%) of *T. spiralis* ML incubated with different concentrations of ALN and ABZ over a 96-h period

Larva			1 h	3 h	6 h	12 h	24 h	48 h	72 h	96 h
Drug-free Control		Mean ± SD	0^a^ ± 0	0^a^ ± 0	0^c^ ± 0	0^e^ ± 0	1.3^e^ ± 0.58	3^e^ ± 1	5.7^e^ ± 1.2	8.7^d^ ± 0.58
		MR	0%	0%	0%	0%	4.3%	10%	19%	29%
DMSO Control		Mean ± SD	0^a^ ± 0	0^a^ ± 0	0^c^ ± 0	0^e^ ± 0	1.7^e^ ± 0.58	3.3^e#^ ± 0.58	6.3^e^ ± 0.58	9.3^d^ ± 0.58
		MR	0%	0%	0%	0%	5.6%	11%	21%	31%
ALN (µg/ml)	3.1	Mean ± SD	0^a^ ± 0	0^a^ ± 0	0^c^ ± 0	0^e^ ± 0	1.7^e^ ± 0.58	5.3^d#^ ± 0.58	13.3^d^ ± 0.58	19^c^ ± 1
		MR	0%	0%	0%	0%	5.6%	17.6%	44.3%	63.3%
	6.2	Mean ± SD	0^a^ ± 0	0^a^ ± 0	0^c^ ± 0	0^e^ ± 0	7.3^d^ ± 0.58	12^c#^ ± 1	16.7^c^ ± 0.58	22.7^b^ ± 0.58
		MR	0%	0%	0%	0%	24.3%	40%	55.6%	75.6%
	12.4	Mean ± SD	0^a^ ± 0	0^a^ ± 0	0^c^ ± 0	2.7^d^ ± 0.58	13^c^ ± 1	20^b#^ ± 1	24.7^b^ ± 0.58	28.7^a^ ± 0.58
		MR	0%	0%	0%	9%	43.3%	66.6%	82.3%	95.6%
	24.8	Mean ± SD	0^a^ ± 0	0^a^ ± 0	0^c^ ± 0	5^c^ ± 1	22^b^ ± 1	28.7^a#^ ± 0.58	30^a^ ± 0	30^a^ ± 0
		MR	0%	0%	0%	16.6%	73.3%	95.6%	100%	100%
	49.6	Mean ± SD	0^a^ ± 0	0^a^ ± 0	0^c^ ± 0	9^ab^ ± 1	24.7^a^ ± 0.58	30^a#^ ± 0	30^a^ ± 0	30^a^ ± 0
		MR	0%	0%	0%	30%	82.3%	100%	100%	100%
ABZ (µg/ml)	25	Mean ± SD	0^a^ ± 0	0^a^ ± 0	2.3^b^ ± 0.58	7.7^b^ ± 0.58	24^ab^ ± 1	29^a#^ ± 0	30^a^ ± 0	30^a^ ± 0
		MR	0%	0%	7.6%	25.6%	80%	96.6%	100%	100%
	50	Mean ± SD	0^a^ ± 0	0^a^ ± 0	6^a^ ± 1	10.3^a^ ± 0.58	25.7^a^ ± 0.58	30^a#^ ± 0	30^a^ ± 0	30^a^ ± 0
		MR	0%	0%	20%	34.3%	85.6%	100%	100%	100%
p-value			–	–	< 0.001^**^	< 0.001^**^	< 0.001^**^	< 0.001^**^	< 0.001^**^	< 0.001^**^

*MR* mortality rate

Mean number of larvae in each group is 30

Three replicas for each group. Data was expressed using Mean ± SD

F: F for One way ANOVA test, pairwise comparison between each 2 groups were done using Post Hoc Test (Tukey)

Different small letters^(a−e)^ indicate significant difference at (p ≤ 0.05) between study groups

Similar small letters^(a−e)^ indicate non-significant difference between study groups

p-value > 0.05 is non-significant, *p-value ≤ 0.05 is statistically significant, ** p-value < 0.001 is highly significant

Noteworthy, the potential anti-parasitic of the bisphosphonates has been attributed to their inhibitory effects on the mevalonate pathway, that required for the synthesis of essential isoprenoids in eukaryotes. Isoprenoids are necessary for the parasite growth and viability, besides the integrity and function of their cell membrane and organelles, and other processes involving cellular signaling. In particular, bisphosphonates block the mevalonate pathway's rate-limiting enzyme, farnesyl pyrophosphate synthase (FPPS) [57]. Moreover, Branco Santos et al. [60] proved that the disturbance of this metabolic pathway can lead to toxicity, changes in the cells function, structure and a loss of homeostasis.

Additionally, incubation of *T. spiralis* AW and ML for 6 h with ABZ revealed lethal effects with mortality rates of gradual increase. Briefly, the first 48 h of incubation with 25 μg/mL ABZ prompted dead for 73.3% of AW and 96.6% of ML, furthermore, 50 μg/mL of ABZ produced 100% dead for both AW and ML (Tables 2 & 3). Albendazole has been reported previously to disrupt the functions of microtubules in both mammalian cells and parasites, through the inhibition of *ß*-tubulin polymerization into microtubules, which in turn inhibits the glucose uptake, transport and eventually causes a glycogen shortage in the parasites [61]. Furthermore, ABZ inhibits malate dehydrogenase, Krebs cycle, with a subsequent reduction in ATP generation. The thing that results in energy exhaustion, the parasite's immobilization and ultimate death [62].

Based on the current data, the mortality rates in *T. spiralis* adults and larvae significantly increased upon treatment with ALN at 24.8 and 49.6 μg/mL after incubation for 48 and 72 h respectively. However, no significant increase in the mortality rates was recorded for ABZ at 25 and 50 μg/mL (Figs. 1 & 2).

### Effect of ALN and ABZ on ultrastructure of *T. spiralis* AW and ML

The tegument of helminths serves as a barrier keeping the parasite safe from the unsuitable surrounding environmental conditions. To this end, Abou Hussien et al. [63] proved that the tegument is essential for nutrients absorption, immune protection, osmoregulation and support of the parasite’s structure. Furthermore, the primary mode of action of anthelmintic drugs is the disruption of the parasite's metabolism and/or the destruction of its cuticle and cytoskeleton, which ultimately results in paralysis and death [64]. Accordingly, the viability outcomes for ALN (24.8 and 49.6 μg/mL) supported the need to evaluate its effect on the ultrastructure of *T. spiralis* AW and ML, in comparison with the blank control and the reference ABZ at concentrations of 25 and 50 μg/mL.

**Fig. 3:**
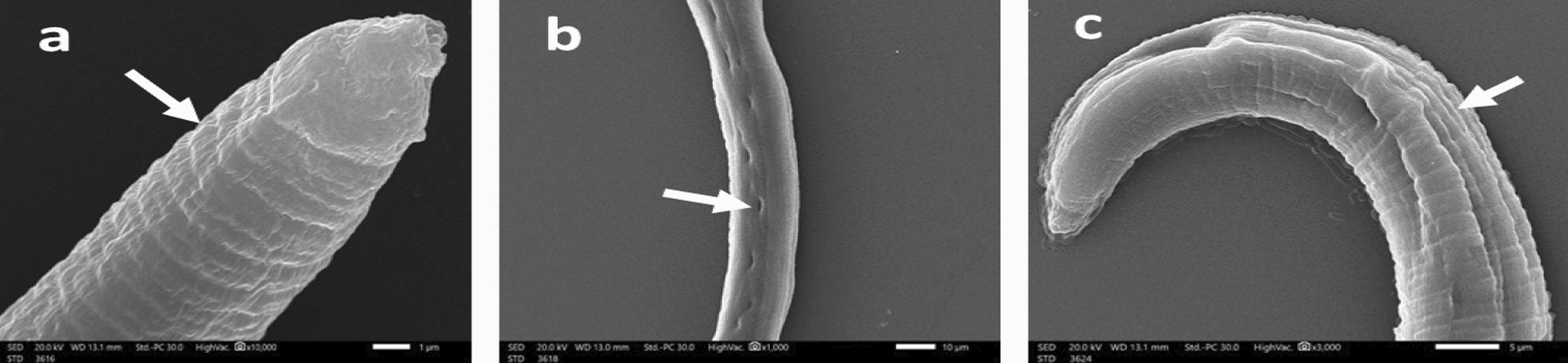
SEM of *T. spiralis* adult worms of the drug-free control group after 48 h incubation in RPMI-1640 medium showing (**a**): normal architecture of the cuticle annulations and transverse ridges (× 10,000), (**b**) normal appearance of hypodermal glands’ openings (× 1000), and (**c**): tapering anterior end with normal longitudinal ridges (× 3000)

Generally, 48 h post-incubation of drug-free control containing *T. spiralis* AW demonstrated normal architecture of the cuticle with normal transverse cuticular annulations, longitudinal ridges, normal appearance of hypodermal glands’ openings and tapering anterior end as detected by scanning electron microscopy (Fig. 3).

**Fig. 4:**
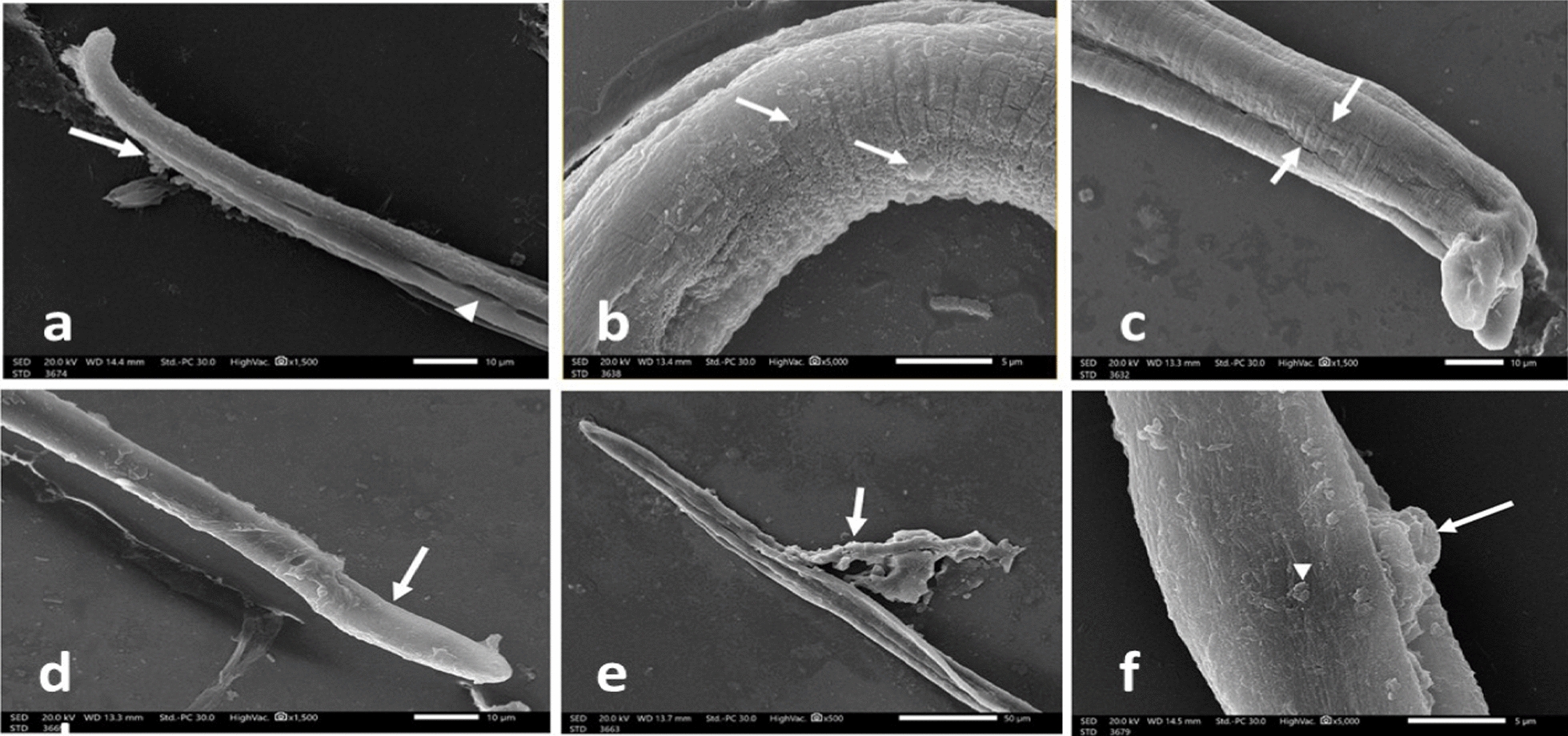
*T. spiralis* adult worm incubated in RPMI-1640 medium containing ALN (24.8 μg/ml) for 48 h showing (**a**): loss of the normal architecture of the cuticle, flattening of the cuticle annulations and widening of the hypodermal glands’ openings (× 1500), (**b**): blebs formation (× 5000), and (**c**) appearance of fissures in the cuticle (× 1500). *T. spiralis* adult worm incubated in RPMI-1640 medium containing ALN (49.6 μg/ml) for 48 h showing (**d**): complete loss of annulations (× 1500), (**e**) with sloughing of large areas of the cuticle (× 500), and (**f**): appearance of multiple vesicles (arrowhead) and a large cauliflower mass (arrow) (× 5000)

**Fig. 5:**
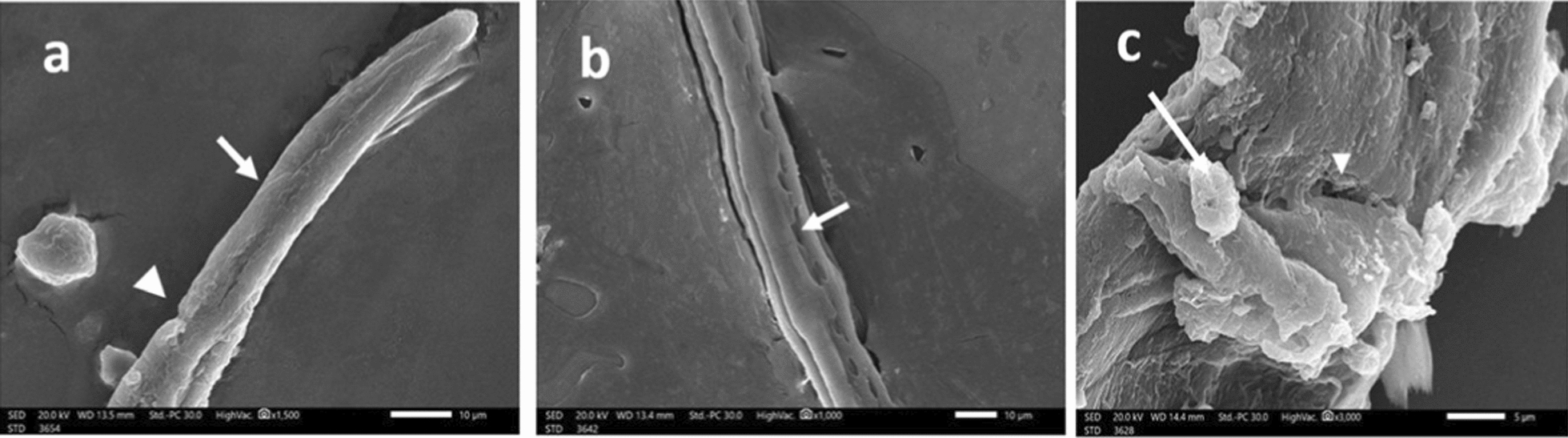
*T. spiralis* adult worm incubated in RPMI-1640 medium containing ALB (25 μg/ml) for 48 h showing: **a** focal destruction in the cuticle (arrowhead) and flattened cuticular annulations (arrow) (× 1500), **b** widened hypodermal glands’ openings (× 1000). *T. spiralis* adult worm incubated in RPMI-1640 medium containing ALB (50 μg/ml) for 48 h showing (**c**): prominent sloughing of wide areas of the cuticle (arrowhead) (× 500), large cauliflower mass (arrow) and multiple blebs (× 5000)

In contrast, 48 h post-incubation of the AW with 24.8 μg/mL of ALN (Figs. 4a, b, & c) or 25 μg/mL of ABZ (Fig. 5a & b), established various degrees of destruction, including loss of the normal architecture of the cuticle with swelling of the anterior end, flattening of the cuticle annulations, and widening of the hypodermal glands’ openings. In addition, fissures in the cuticle were observed with the appearance of some blebs. Moreover, higher concentrations as 49.6 μg/mL of ALN (Fig. 4 d, e, & f) or 50 μg/mL of ABZ (Fig. 5c) revealed severe destruction with sloughing of large areas of the cuticle and complete loss of annulations with the appearance of multiple vesicles and large cauliflower masses.

**Fig. 6:**
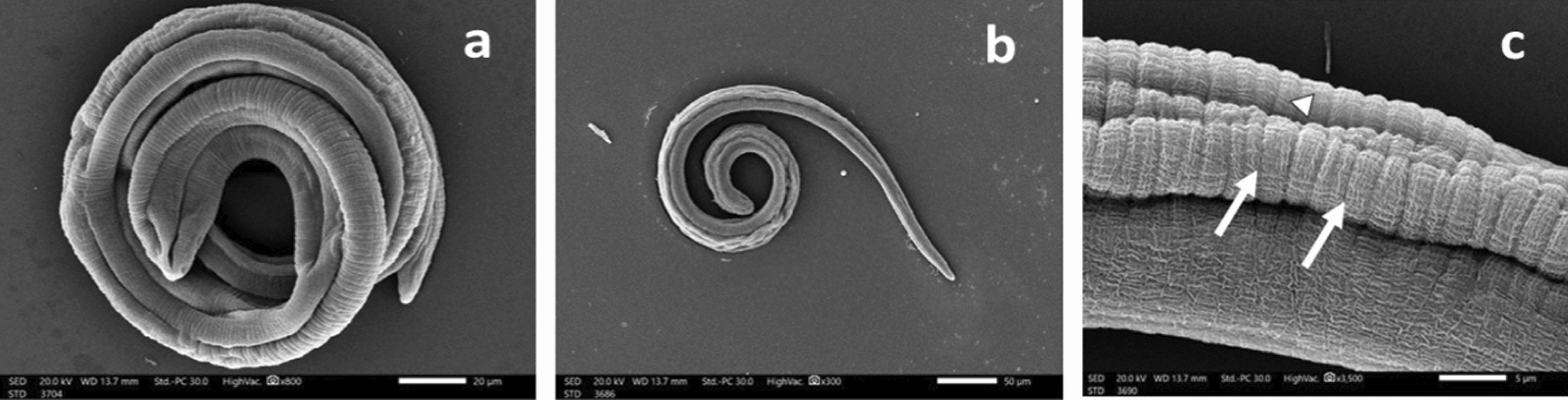
*T. spiralis* larva after 48 h incubation in drug-free RPMI-1640 medium showing: (**a**) normal comma-shaped larva with intact cuticular folds (× 800) and (**b**) coiled posterior end (× 300). (**c**) Normal architecture of the cuticle with transverse creases (arrow) and longitudinal ridges (arrowhead) (× 3500)

**Fig. 7:**
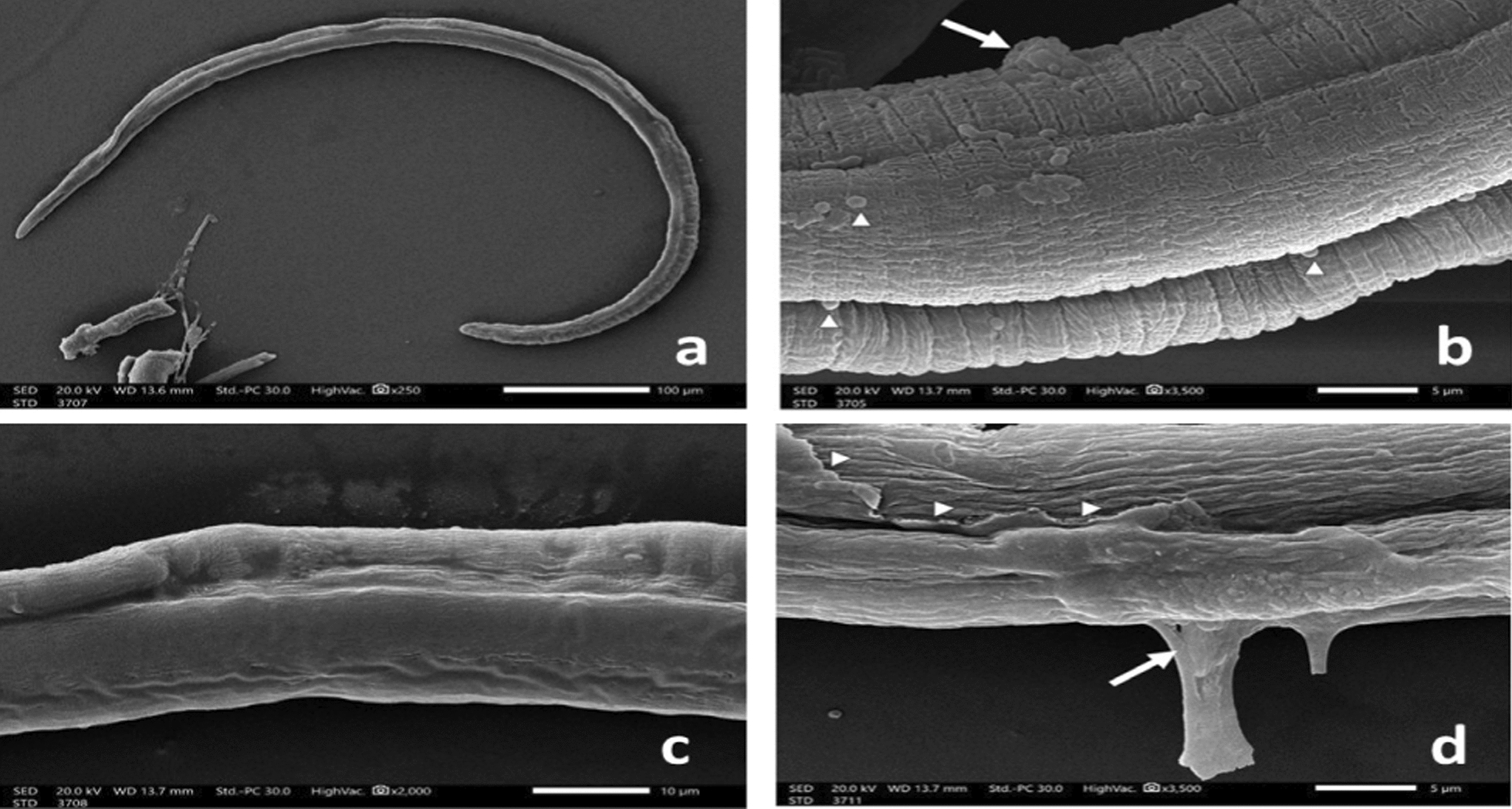
*T. spiralis *larva after 48 h incubation in RPMI-1640 medium containing ALN (24.8 μg/ml) showing: **a** loss of normal coiling of the larva (× 250). **b** Flattening of the cuticle annulations and transverse ridges with appearance of some blebs (arrowhead) and small cauliflower mass (arrow) (× 3500). *T. spiralis* larva incubated in RPMI-1640 medium containing ALN (49.6 μg/ml) for 48 h showing: (**c**) diffuse cuticular swelling with loss of annulation and longitudinal ridges (× 2000). **d** Destruction (arrowhead) and sloughing (arrow) of the cuticle (× 3500)

**Fig. 8:**
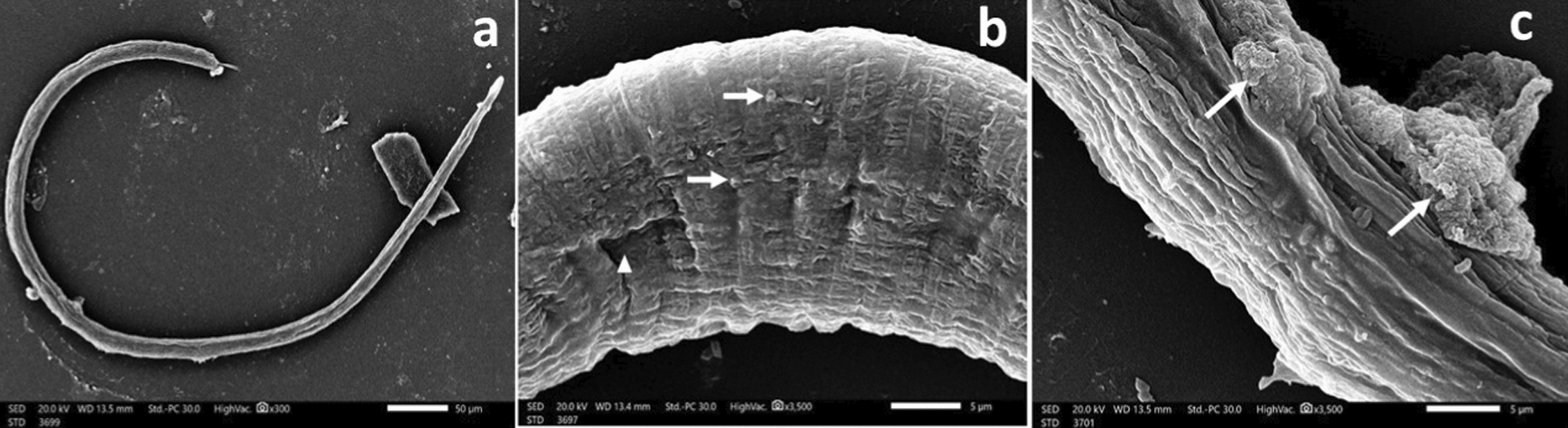
*T. spiralis* larva after 48 h incubation in RPMI-1640 medium containing ALB (25 μg/ml) showing: **a** loss of the posterior coiling(× 300). b Distorted cuticular architecture with focal destruction of the cuticle (arrowhead), with the appearance of small blebs (arrow) (× 3500). **c** *T. spiralis* larva incubated in RPMI-1640 medium containing ALB (50 μg/ml) for 48 h showing multiple cauliflower masses (arrow) with destruction of the cuticle (× 3500)

*T. spiralis* ML after 48 h incubation in drug-free medium (blank control) (Fig. 6) displayed a normal comma-shaped appearance with a coiled posterior end, the normal architecture of non-treated ML showed cuticular transverse creases and longitudinal ridges. Incubation of ML with either ALN (24.8 μg/mL) Figs. 7a & b or ABZ (25 μg/mL) Figs. (8a & b), induced loss of normal coiling with mild to moderate deformities in the cuticle annulations and transverse ridges with the appearance of some blebs and small vesicles. Higher concentrations of ALN (49.6 μg/mL) Figs. (7c & d) and ABZ (50 μg/mL) (Fig. 8c) were associated with diffuse swelling of the cuticle with loss of annulation and transverse ridges in large areas in the cuticle. Sloughing of large areas of the cuticle was observed and the appearance of large cauliflower masses.

This was in agreement with El-Sayad et al. [40], who observed that nifedipine-induced tegument destruction in in vitro treated AW and ML with apparent multiple degenerative changes, including the appearance of blebs, multiple vesicles, fissures and loss of normal annulations. In the same context, progesterone and mifepristone were identified as promising candidates against trichinellosis owing to their significant effect on the parasite’s tegument [65].

Martin [66] explained that helminths tegument alterations are thought to be a reliable predictor of a drug’s potential anthelmintic action. Furthermore, blebbing was reported to form as the parasite tried to repair the damaged surface membrane as a result of the drug's effects. Furthermore, degenerative changes in the cuticle of *T. spiralis* AW and ML were reported with the use of multiple medicinal herbs.

For instances, El-Sayad and co-workers [40] observed the appearance of multiple swellings, large blebs, fissures and vesicles, in addition to sloughing of some areas of the tegument of AW and ML treated with *Chrysanthemum coronarium* extract. In the same context, Mohammed et al. [64] declared that *B. indica* extract was able to induce *T. spiralis* AW shrinking and sloughing at some areas of the cuticle and marked cuticular destruction, and confirmed this via in silico study. Similarly, Fahmy et al. [67] reported the destruction of the tegument with the use of clove oil (*Syzygium aromaticum)* against AW and ML of *T. spiralis*. Generally, the lack in the characterization of the potential targets for ALN against *T. spiralis* calls the need for exploring the possible mechanisms of action acting against the parasite via the help of a detailed in silico study.

### In silico results

#### Selection of the targeted receptor proteins of *T. spiralis*

After being ingested, *T. spiralis* ML proceed to IL, then invades the host’s small intestinal epithelium and undergoes four molting to develop to adult worms (AW) in 31 h post-infection, followed by intestinal mucosa invasion [68]. During the intestinal stage of *T. spiralis* infection, the excretory-secretory (ES) antigens produced by the AW result in early exposure to the host’s immune system and elicit the production of specific anti-Trichinella antibodies. *T. spiralis* surface proteins (*i.e., Ts*-SP, *Ts*-PPase, *Ts*-MAPRC2, *Ts*-TS, *Ts*-MIF, etc.) may play important function in the larval invasion and the development process. Their analysis could provide useful information help in the elucidation of the host-parasite interaction, identify the early diagnostic antigens and the targets for vaccine [69].

Briefly, Todorova and Stoyanov [70] showed that, the proteinases secreted from *T. spiralis* AW had catalytic hydrolase activity and degrading fibrinogen and plasminogen. *Ts*-serine proteinases in AW were present in E–S products and the purified enzymes displayed enzymatic activity; hence, it might be the invasion-related proteins and so are good targets for vaccine. Furthermore, inorganic-pyrophosphatase in *T. spiralis* (*Ts*-PPase) was reported by Hu et al. [71], as an important participant in energy cycle and plays a vital role in hydrolysis of inorganic pyrophosphate (PPi) into inorganic phosphate (Pi), thus, its inhibition can result in the suppression of AW and newborn larvae development. Moreover, Aleem and co-workers [72] proved that the inhibition of *T. spiralis* membrane-associated progesterone receptor (*Ts*-MAPRC2), led to the reduction of the worm burden, and therefore, suggested that, *Ts*-MAPRC2 might be a novel molecular target useful for the development of vaccines against *T. spiralis* infection. Besides, the housekeeping gene, thymidylate synthase (*Ts*-TS), that plays an important role in DNA synthesis and ubiquitous phyletic distribution via binding its own mRNA and repress translation. The expression level of thymidylate synthase in *T. spiralis* was similar in ML, AW and newborn larvae, and therefore, a good target for its inhibition [73].

Parasites normally possess the ability to escape from host immune attack, by secreting a homolog of host MIF, that has the capability of modifying the activity of human monocytes–macrophages [74]. Moreover, MIF recombinant protein inhibited migration of human peripheral blood mononuclear cells, recently, it has been discovered that MIF not only plays a critical role in inflammation but also has endocrine and enzymatic function. The MIF gene is expressed in various developmental stages of *T. spiralis*, including AW, newborn larvae and ML.

Recently, Mohammed et al. [64] reported that the cysteine protease (*Ts*-CF1), which is expressed by *T. spiralis* at all life stages and localized in the cuticle and stichosome, is a potential drug target and/or vaccine candidate against *T. spiralis* infection.

### Homology modeling, optimization and validation

Protein fold recognition Server was conducted to build the 3D structures for *Ts*-CF1, serine proteinases, *Ts-*PPase and *Ts*-MAPRC2. Resulted templets recorded overall sequence similarities; for *Ts*-CF1 with 53% (the crystal structure of human cathepsin F ´1M6D`), *Ts*-serine proteinases with 60% (the crystal structure of conserpin in the latent state ´5cdz`), *Ts*-PPase with 76% (the crystal structure of human inorganic pyrophosphatase ´6C45`), and *Ts*-MAPRC2 with 47% (the crystal structure of human pgrmc1 cytochrome b5-like domain ´4xy8`). Post-minimization using YASARA, the generated models were assessed using SAVES. The x-axis of the Ramachandran plot is split into four quadrants include regions of the low-energy, the allowed, the generally allowed and the disallowed. PROCHECK revealed the percent of the residues that fell in the most favored regions of the Ramachandran plot are as follow; *Ts*-PPase (85.1%), *Ts*-CF1 (87.2%), *Ts*-MAPRC2 (87.5%) and *Ts*-serine proteinase proteins (87.6%), however, percents of 14.1%, 12.8%, 9.6% and 9.4% fell in the additional allowed regions, respectively, in addition, values of 0.8%, 0.0%, 2.9% and 2.1% fell in the generously allowed regions, respectively, furthermore, no residue was in the disallowed regions for all generated models except for *Ts*-serine proteinases recorded only 0.9%, however, it is a part from the active pocket. Additionally, the overall quality factor and compatibility of the atomic model with its amino acid sequence (3D-1D) for all models; *Ts*-PPase, *Ts*-CF1, *Ts*-MAPRC2, and *Ts*-serine proteinase, were observed as 77.74, 98.09, 84.40 and 53.17 respectively. Thus, the Ramachandran plot, ERRAT and PROSA results, confirmed that, the generated models were modeled well enough within the range of a high-quality [75].

### Molecular docking

The analysis predicts the best-pose for drugs ´ABZ and ALN` at the inhibition pocket of *T. spiralis* surface proteins, promising results came out and summarized that, both ligands fit well the active pocket of each receptor and revealed an accepted binging affinities as well as molecular interactions including hydrogen bonding and hydrophobic contacts with the key amino acids at the active site (Table 1). Nevertheless, the highest binding energy reflects the potential inhibition of a drug candidate, the resulted binding affinities recorded for our complexes may not be considered as a determinant property to clarify their activity against the parasite, however, we can rely on their molecular interactions (HB, Hyd) with the target receptors.

**Fig. 9.**
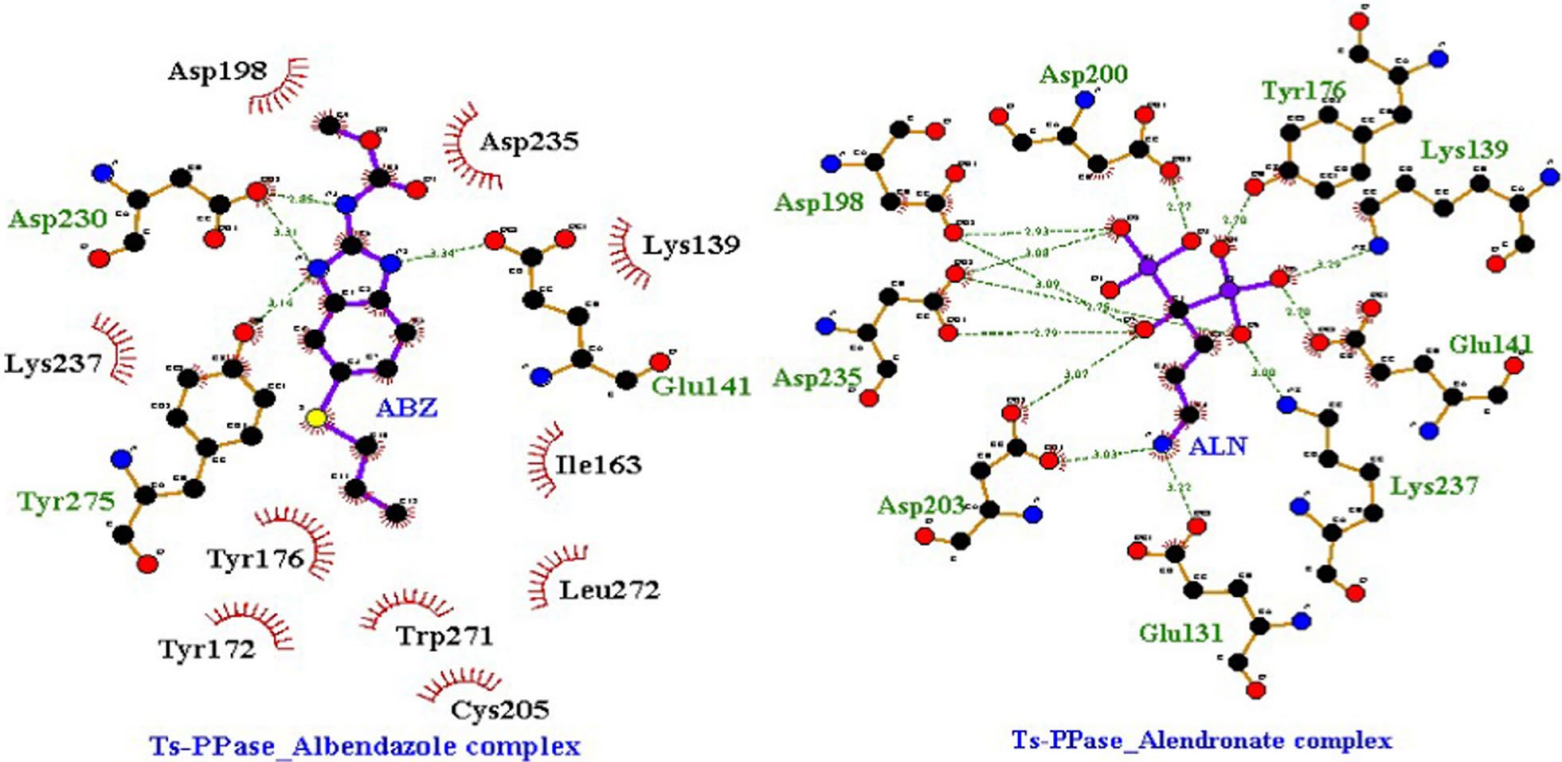
2D binding interactions of ABZ (albendazole) and ALN (alendronate) with *Ts*-PPase. The hydrogen bonds are represented as dotted bonds, while the hydrophobic interactions are represented as red rays

Briefly, as demonstrated in (Table 1) that ABZ recorded the highest binding affinity, whereas, the highest number of molecular interactions was achieved by ALN. Concisely, the highest binding energy recorded for ALN was –5.5 kcal/mol compared to −6.8 kcal/mol for ABZ, however, 9 HB-interactions with *Ts*-PPase key residues like Glu131, Lys139, Glu141, Tyr176, Asp198, Asn200, Asp203, Asp235 and Lys237 recorded for ALN, compared to 3 H-bonds with Glu141, Asp230 and Tyr275 for ABZ, in addition to 10 Hyd interactions with (Lys139, Ile163, Tyr172, Tyr176, Asp198, Cys205, Asp235, Lys237, Trp271 and Leu272 (Fig. 9).

It is apparent clearly from (Table 1) that both ABZ and ALN targeted *Ts*-serine proteinase and anchored well at the binding cavity with binding affinities of –4.0 and –3.6 kcal/mol respectively, while ALN interacted with key amino acids Glu177 and Tyr219 *vis* HB-interactions and with Tyr180, Arg209, Ser210, Phe222 and Lys273 by Hyd-interactions, however, ABZ interacted with only two key amino acids Lys245 (HB) and with Glu177 (Hyd), together with other residues like Pro179, Glu182, Thr183, Phe185 and Leu246 via Hyd-interactions (**S-1**).

Moreover, the reduction in the worm count of *T. spiralis* was confirmed via the docking results of ALN and ABZ against *Ts*-MAPRC2, definitely, both ligands settled at the binding pocket with affinity binding of –4.3 and –4.9 kcal/mol respectively, however, ALN recorded 5 HB and 2 Hyd-interactions, with key amino acids Asp112, Gly145, Leu146, Ala148, Arg150 and Gln140, along with Met163. Moreover, ABZ showed 1 HB and 6 Hyd-interactions with key amino acids Arg150, Arg109, Asp112, Gly145 and Leu146, along with Gln140 and Gly142 (**S-2**).

Furthermore, Dabrowska and coworkers [73] reported that, targeting thymidylate synthase should led to the inhibition of *T. spiralis* DNA synthesis, this was confirmed via the docking insertion of ABZ and ALN at the active pocket with binding affinities of –6.5 and –4.8 kcal/mol respectively, and with amino acids like Glu81, Leu215 and Asn220 forming H-bonding and Arg72, Phe74, Ile102, Tyr103, Gly216 and Phe219 forming Hyd-interactions (S-3).

Additionally, MIF gene is representing a good target for *T. spiralis* inhibition [74], so, in silico docking of ABZ and ALN revealed that both ligands fit well the active pocket with –6.3 and –4.7 kcal/mol respectively, however, the key residues like Pro1, Tyr36 and Gly107 interacted with ALN via HB, and with ABZ through Hyd-interactions, together with Ile2, Lys32, Ile64, Val106, Gly107, Trp108 and Phe113 (S-4).

Recently, Mohammed et al. [64] reported the in vitro and in vivo inhibition of *T. spiralis* and concluded the mode of inhibition via targeting the cysteine protease (*Ts*-CF1). Consequently, our results cleared out that both ABZ and ALN fit the binding cavity at *Ts*-CF1, with binding affinity – 4.3 kcal/mol and 5 HB-interactions with Gln167, Gly168, Cys170, His309 and Trp335 for ALN, besides, – 5.2 kcal/mol for ABZ with 1 HB-interaction at Gln167, and Hyd-interactions at Gly171, Cys173, Met288 and Trp339 respectively (S-5).

Moreover, *T. spiralis* escapes the adaptive immune attack and facilitates their survival through the secretion of calreticulin protein [76], which make this calreticulin a good target for their inhibition [64]. As tabulated in Table (1), ALN fitted the active pocket forming 8 HB-interactions with the key amino acids (Ser56, His57, Leu60, Asp66, Thr100, Lys102, Thr173 and Asp332) with binding affinity −5.4 kcal/mol. However, ABZ revealed binding affinity of –6.2 kcal/mol and interacted with Asp63 and Gln350 through 2H-bonds, besides, Hyd-interactions with Phe61, Ala62, Gly68, Leu354 and others (S-6).

Our docking findings represented a suggestive overview concerning the potential activity of ALN to inhibit the selected receptors, which is an indicative for its potential anthelmintic activity against *T. spiralis* at different life stages, though one mechanism of action or more.

### Molecular dynamics simulation

Considering that, the conformational changes associated with enzyme inhibition are time dependent, hence, it may be missed in such docking analysis. Thus, the dynamic behavior of a protein–ligand complex over time can be explained through the extensive sampling of the dynamic’s simulation trajectory frames [77, 78].

Molecular dynamics analysis was executed for complexes *Ts*-PPase_ALN and *Ts*-PPase_ABZ at the active pocket of *Ts*-PPase. Complex’s selection was depending on the highest binding affinity and largest number of molecular interactions as obtained from docking analysis. MD simulation analyzed their behavior for a period of 100 ns in the production step, monitoring their structural and dynamics properties including RMSD, RMSF, RG and SASA proved their structural stability and compactness, additionally, the stability and strength of their molecular interactions (HB and Hyd) were investigated via H-bonds and clusters analysis.

### Root-mean square deviation

**Fig. 10:**
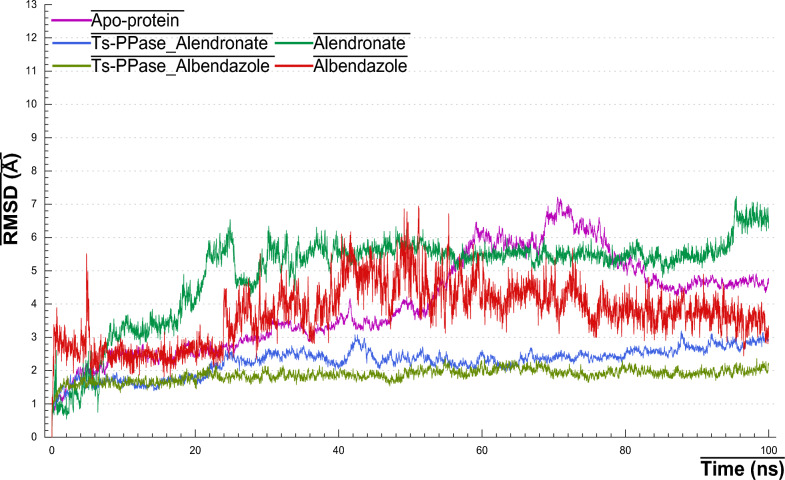
RMSD plots of *Ts*-PPase_ligand complexes and the apo-form during 100 ns MD simulation

The RMSD values of backbone atoms for *Ts*-PPase_ALN, *Ts*-PPase_ABZ and their apo-form were estimated considering the initial frame of 100 ns MD simulation as reference. As detected in (Fig. 10) that both complexes *Ts*-PPase_ALN and *Ts*-PPase_ABZ maintained stable fluctuations within the acceptable range of 2–3 Å throughout 100 ns trajectory, however, their apo-form fluctuated drastically from time 25 ns until it reached 85 ns and then attained equilibrium.

On the other hand, alendronate showed a fluctuating behavior till it reached 30 ns and then stopped fluctuation and held equilibrium with an average RMSD values of 5.61 ± 1.00 Å, which could be a reason for the high number of rotatable bonds, which sometimes forced alendronate to deviate from the active site and attach to another one as reported [79], nevertheless, it has no effect on its molecular interactions with the key amino acids as it will be discussed latter on. However, albendazole started to fluctuate from 25 ns and keep fluctuations till the end of simulation with an average RMSD values of 2.5–6.5 Å. Notwithstanding, comparing to the apo-form we can conclude that all complexes preserved stability throughout the simulation which might be a reason for the presence of the ligand.

### Root-mean square fluctuation

**Fig. 11:**
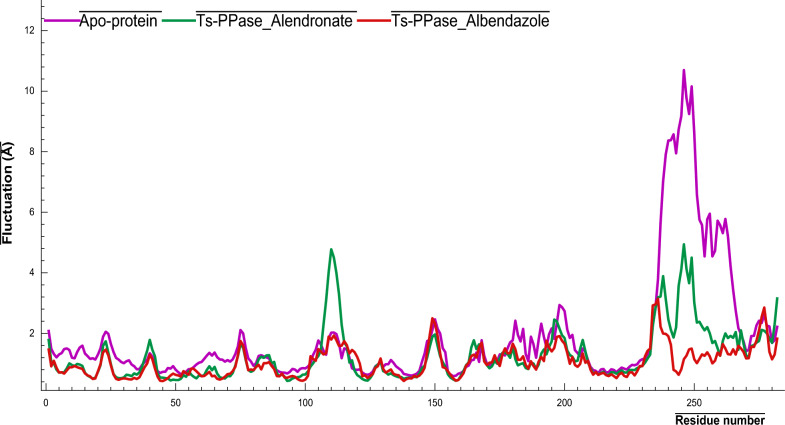
RMSF (Chain Cα) of *Ts*-PPase_ligand complexes and the apo-form during 100 ns MD simulation

RMSF is a determinant of structural flexibility within a molecule [80], thus, lower RMSF degrees reflect higher stability of the protein–ligand complex [81]. The RMSF graph depicted in (Fig. 11) indicated a relatively high level of RMSF at the region that corresponds to residues 100–115, whereas the core structural fold region that contains the active site residues shows lower RMSF values < 2 Å. Therefore, the residues within this region have minimum flexibility and thus have higher stability. However, residues 233–268 of the apo-form recorded their highest fluctuations with 10.5 Å compared to residues 233–250 for *Ts*-PPase_ALN with low fluctuations 4.9 Å, interestingly, *Ts*-PPase_ABZ complex revealed lower fluctuations within the residues 233–238 with 2.9 Å. Noteworthy, the resulted RMSF supports the concept that the formed complexes are stable at the site each ligand binds to and do not significantly change the residue fluctuations at any regions.

### Radius of gyration (RG)

**Fig. 12:**
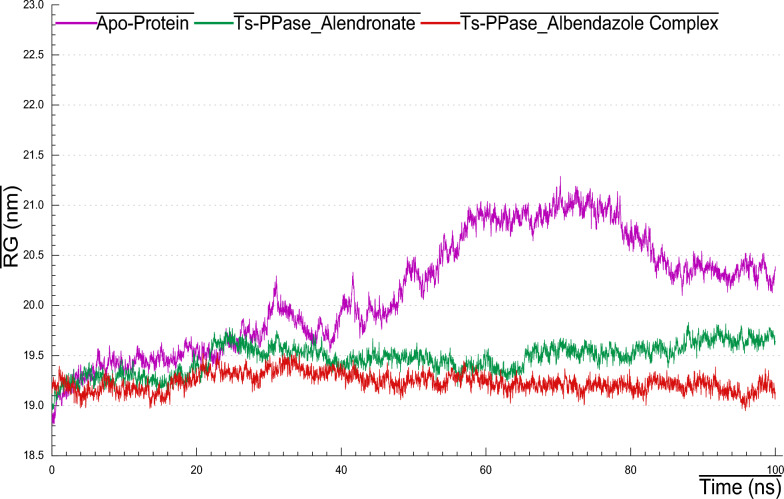
RG plots of *Ts*-PPase_ligand complexes along with the apo-form during 100 ns MD simulation

It is used to determine how is the compactness of a system, which is a determinant of stability. Over 100 ns simulation period, the average RG (Fig. 12) was found to be ranged between 18.9–19.2 nm for *Ts*-PPase_ALN and 18.9–19.6 nm for the apo-form, however, RG of *Ts*-PPase_ABZ revealed 18.9–21.1 nm. The lower values of RG indicated that the ligand binding to the protein's active site does not induce major conformational changes in the protein structure, and the resulted complex became tighter and more compact. This suggests that *Ts*-PPase_ALN and *Ts*-PPase_ABZ complexes remained stable throughout the entire simulation period.

### Solvent accessible surface area (SASA)

**Fig. 13:**
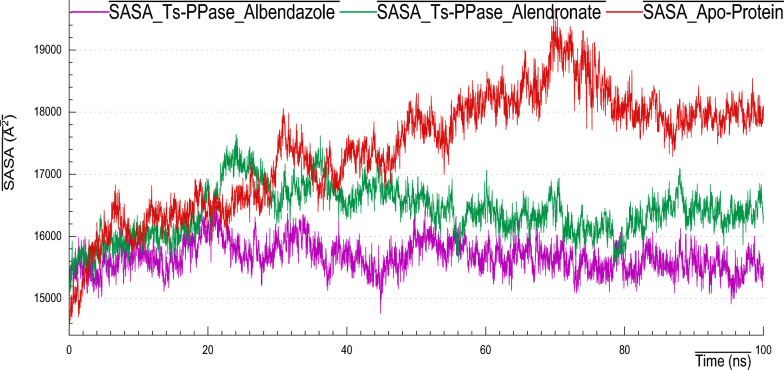
SASA plots of *Ts*-PPase_ligand complexes and their apo-form during 100 ns MD simulation

A crucial and pivotal parameter in protein structure analysis is SASA, it is the study of the influence of the surrounding solvent on a complex. It provides insights into the protein’s folding, stability and interactions with other molecules. Here, as we can observing (Fig. 13) the plots of SASA values expressed in (Å^2^), it is clearly appeared that, an improvement in SASA values was observed and was attributed to the ligand binding. Concisely, SASA of the apo-form gave 15.500–19.000 Å^2^, however, for *Ts*-PPase_ALN and *Ts*-PPase_ABZ the SASA values ranged from 15.500–16.500 Å^2^, which accounted for their compactness upon ligand binding and therefore folding and stability.

### Hydrogen bonding

**Fig. 14:**
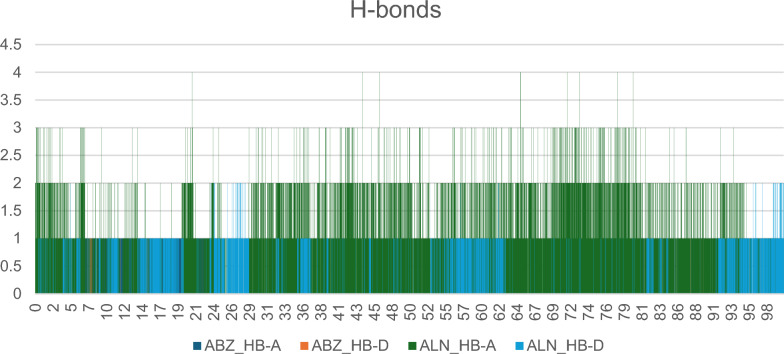
Hydrogen bond donor and acceptor as resulted for ALN and ABZ

The analysis of the H-bonding (Fig. 14) established both donor and acceptor hydrogen bonds and their occupancy rates (**S-7**). Putting into account that, the occupancy rates refer to the time period for H-bonds to be detected in a single trajectory throughout the MD simulation. Hence, occupancy rats with higher values refer to the major of H-bonding in system stability [80]. The overall occupancy rate recorded for ALN was 86.51%, however, it scored 19.46% for ABZ. Careful inspection demonstrated that the highest values of occupancy were recorded to be 44.70% and 14.38%, and refer to the interaction of ALN with residues like Asp200 and Asp203 respectively, moreover, ABZ revealed 11.14% occupancy rate accounted for the interaction with Asp203. Collectively, these observations are suggestive for how importance is the H-bonding in stabilizing the *Ts*-PPase_ALN and *Ts*-PPase_ABZ complexes.

### Cluster analysis and the behavior of H-bonding

**Fig. 15:**
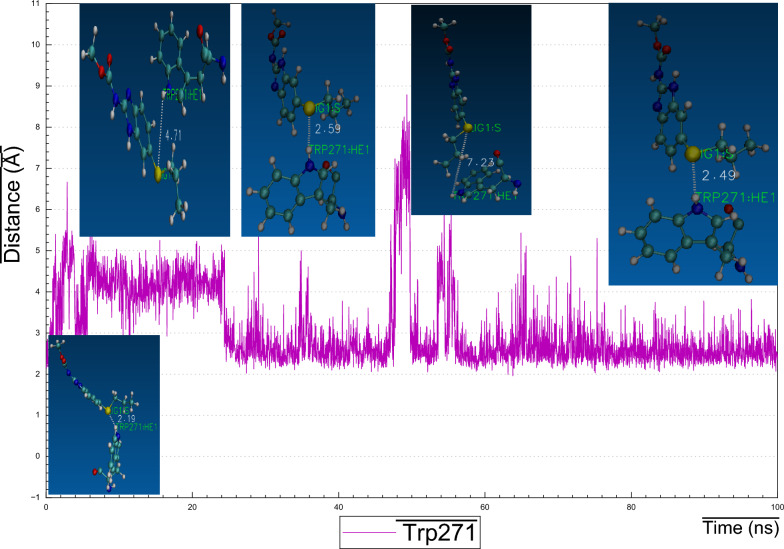
Distance plots of atom pairs involved in the formation of hydrogen bond between albendazole and Trp271 as extracted by cluster analysis during the course of MD simulation trajectory. Note down that all the hydrogen bonds of albendazole with Asp230, Tyr275 and Glu141 are unstable throughout simulation. However, Trp271 tended to form the relatively stable hydrogen bond throughout the simulation

The structural pose of *Ts*-PPase_ABZ complex resulted from docking analysis was visually inspected, and demonstrated that, ABZ might form four hydrogen bonds with *Ts-*PPase residues, concisely, the hydroxyl hydrogen at the backbone of Asp230 formed two H-bonds with the nitrogen atom of the carbamate side-chain and benzimidazole ring of ABZ, moreover, Tyr275 benzo-hydroxyl hydrogen formed H-bond with the nitrogen atom at the benzimidazole ring, in addition, Glu141 carbonyl oxygen at the backbone formed H-bond with amino hydrogen of benzimidazole ring. However, the resulted best-representative-pose from MD cluster indicated that, the H-bonds formed with Asp230, Tyr275 and Glu141 were not stable enough for longer time simulation, moreover, one new H-bond formed between the sulphur atom of the propylsulfanyl side chain of ABZ and the hydrogen atom substituted on the indole ring of Trp271. After monitoring and plotting the distance between residue Trp271 and ABZ, it became evident that, the new recognized H-bond was relatively stable and consistent with high fluctuations at different time intervals *i.e.,* from 5 to 22 ns (~ 4.71 Å), from 25 to 48 ns (~ 2.59 Å), from 48 to 55 ns (~ 7.23 Å) and then attained equilibrium with distance of (~ 2.49 Å) until the end of the simulation trajectory (Fig. 15).

Interestingly, docking analysis conducted for *Ts*-PPase_ALN system produced a relatively complex network of polar H-bond interactions; the phosphor-hydroxyl hydrogen of ALN formed HB with the carbonyl oxygen of Asp198, Asp200, Asp230 and Asp235. Moreover, the amino-hydrogen on the backbone of Lys139 and Lys237 formed HB with sulphonic oxygen atom. Additionally, the amino-hydrogens at the side chain of ALN formed HB with Asp203 and Glu131 carbonyl oxygen. Finally, Tyr176 benzo-hydroxyl hydrogen formed HB with phosphoryl oxygen of ALN.

**Fig. 16:**
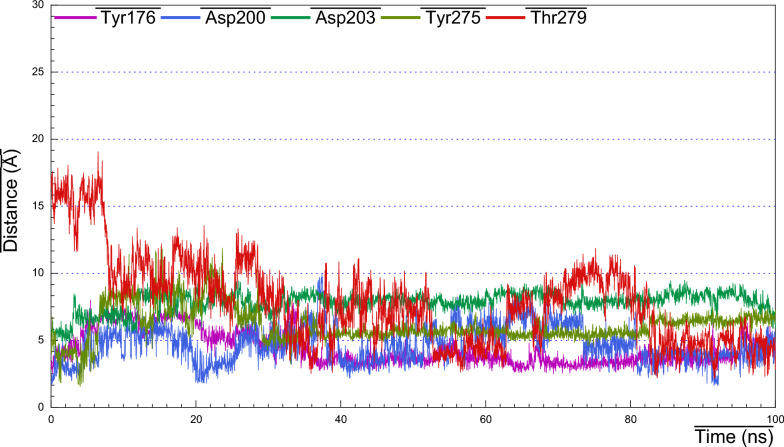
Distance plot of atom pairs involved in the formation of hydrogen bonds between alendronate and Asp200, Asp203, Tyr176, Tyr275 and Thr279 during the course of MD simulation trajectory

As we realized former that, the RMSF plot of *Ts-*PPase_ALN showed relatively low fluctuations at residues 233–251, which might influence the molecular interactions within the key amino acids at the pocket site. Surprisingly, cluster analysis of MD simulation trajectory revealed, preserved polar interactions like the H-bonding between the carbonyl oxygen of Asp200 and Asp203 with the sulphonyl hydrogen of ALN, besides, the benzo-hydroxyl hydrogen of Tyr176 and 275 formed H-bond with the phosphoryl oxygen of ALN, moreover, the carbonyl oxygen of Thr279 formed H-bond through the interaction with the terminal amino hydrogen of ALN (Fig. 16). In addition to the hydrophobic interactions of ALN with Glu233, Asp235 and Lys276 (Fig. 21).

**Fig. 17:**
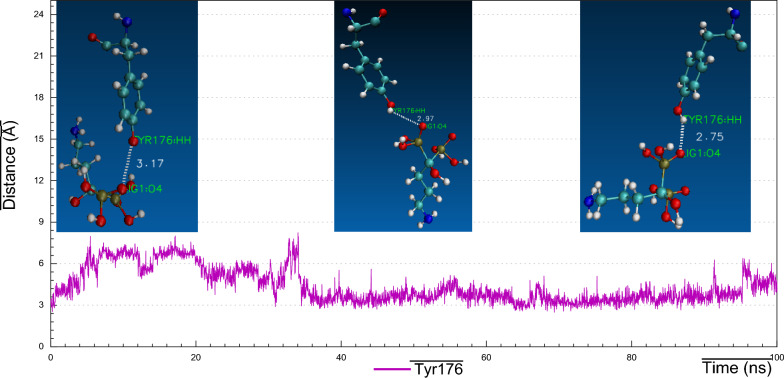
Visual representations of hydrogen bonds between hydroxyl group of alendronate with side chain of Tyr176 at initial, middle and final time frames in MD simulation trajectory

**Fig. 18:**
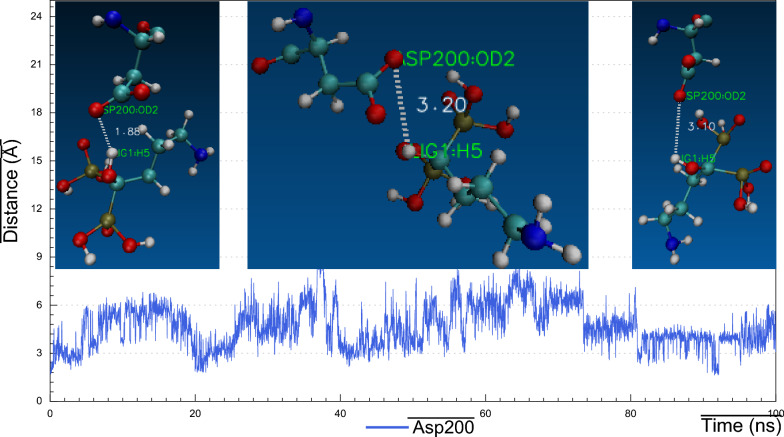
Visual representations of hydrogen bonds between hydroxyl group of alendronate with side chain of Asp200 at initial, middle and final time frames in MD simulation trajectory

**Fig. 19:**
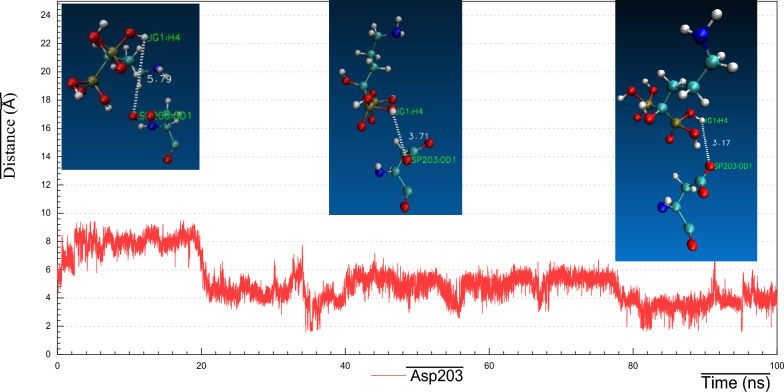
Visual representations of hydrogen bonds between hydroxyl group of alendronate with side chain of Asp203 at initial, middle and final time frames in MD simulation trajectory

These findings were validated by analyzing the distance plots between the corresponding atoms, somehow through, capturing time frame conformations of ALN at different fluctuating intervals to clarify the binding poses. Concisely, distance analysis clearly indicated that, the H-bond formed between Tyr176 and ALN recorded distance 3.17 Å at 5 ns, however, instant fluctuations resulted in bond distance increase with 6.90 Å, and then at ~ 20 fluctuations vanished and it acquired equilibrium with strong H-bond distance of 2.75–2.97 Å till the simulation ended (Fig. 17). Furthermore, the H-bonding between of Asp200 and ALN showed distance of ~ 1.9 Å, followed by fluctuations that increased bond distance with 3–6 Å till 80 ns, then approached equilibrium with stronger H-bond of distance 3.1 Å (Fig. 18). In addition, Asp203 started with bond length ~ 5.5–6.1 Å for the first 20 ns, then with conformational changes, Asp203 moved with a very close vicinity toward ALN, so that, Asp203 formed strong H-bond with distance ~ 2.5–3.5 Å at time span 80 ns (Fig. 19).

**Fig. 20:**
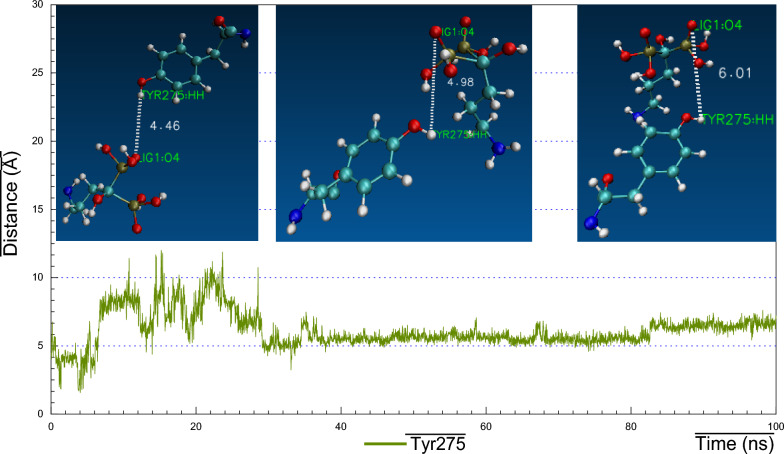
Visual representations of hydrogen bonds between hydroxyl group of alendronate with side chain of Tyr275 at initial, middle and final time frames in MD simulation trajectory

Similar results were also observed for Tyr275 with ALN the formed H-bond began with ~ 4.5 Å distance, then fluctuated through the first 30 ns, after that, it held equilibrium with bond distance ~ 5–6 Å till the end of simulation. Additionally, Thr279 formed weak H-bond with ALN that fluctuated at different time spans and then gained steady at 80 ns with bond distance ~ 5 Å (Fig. 20).

Consequently, the amino acid residues Lys176, Asp200, Asp203, Lys275 and Thr279 retained their H-bond interactions mutually together with other hydrophobic contacts, in such a way to prevent ALN from moving outside the pocket cavity throughout the bulk of the simulation time.

**Fig. 21:**
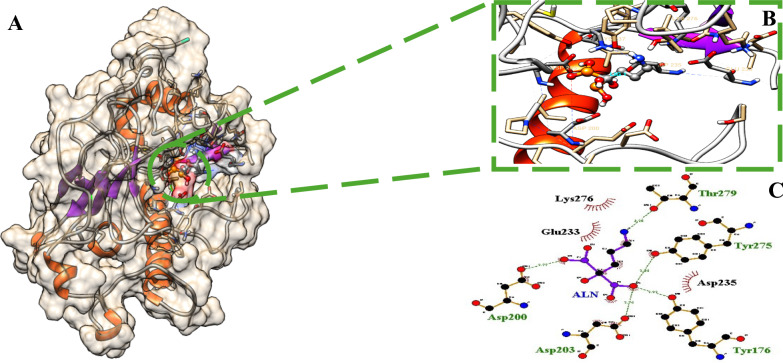
Representation of alendronate in the active pocket of *Ts*-PPase, **A**) the protein surface is represented in pale brown, **B**) the ligand is hosted at the active site in gray,** C**) the representative structure of alendronate from cluster analysis, the hydrogen bonds are represented as dotted bonds, while the hydrophobic interactions are represented as red rays

It is apparent clearly (Fig. 21) that, ALN with the highest H-bond occupancy (86.51%) maintained multiple stronger interactions with the key amino acids during the entire simulation, these interactions could be attributed to the amino-bisphosphonate functional groups [80], moreover, most of the recorded amino acids from docking pose held their interactions with ALN during the entire course of MD simulation, however, in case of ABZ was not, the fact that reflected the ability of ALN to firmly held at the inhibition site of *Ts*-PPase, the thing that accounted for its potential ani-*T. spiralis* activity. Hence, the stronger the interactions, the more the ligand will affect the physiological function of the target proteins; therefore, ligands that bind strongly to the target protein are selected as drug candidates. Based on the molecular docking and molecular dynamics simulation results, the mode of action for ALN as anti-*T. spiralis* could be attributed to its ability to bind the key amino acids at the active pocket of *Ts-*PPase, and hence, inhibits the hydrolysis of inorganic pyrophosphate (PPi) into inorganic phosphate (Pi) which is a vital participant in parasite’s energy cycle, as it can be coupled to several energy demanding biochemical transformations such as DNA replication, protein synthesis and lipid metabolism, and plays an important role in development and molting process of intestinal *T. spiralis* larval stages.

Concisely, our findings here, along with, the reported anti-inflammatory and immunomodulatory properties of ALN [24] may help reduce muscular complications associated with trichinellosis. Additionally, its inhibitory effects on angiogenesis could prove beneficial by targeting muscle larvae and preventing the formation of nurse cells [25].

Interestingly, ALN could be of dual utility that influence patient outcomes, briefly, patients with osteoporosis or other bone disorders who become infected with *T. spiralis* may benefit from ALN’s dual effect, providing therapeutic synergy by targeting both the infection and the underlying bone condition. Moreover, future studies could investigate whether patients on regular use of ALN can have a prophylaxis against infection. A more comprehensive answer to this question could be more clearly elaborated following the validation of the in vitro study results through future experimental in vivo studies.

## Conclusion

According to the current study, ALN exhibited strong in vitro efficacy against adult worms and muscle larvae of *T. spiralis*, possibly because of its obvious impact on the parasite's survival. This outcome was further corroborated by the prominent induced damage to the parasite's tegument, which manifested as fissuring of the cuticle, widening of the hypodermal glands, flattening of the cuticular annulations, and the formation of multiple vesicles and large cauliflower masses. In order to emphasize our findings, we performed comprehensive molecular docking and molecular dynamic studies, from which, it has appeared clearly that the tested drugs, albendazole and alendronate, have shown promising results against various targets of *Trichinella spiralis*, through variety modes of action. In addition, MD simulation analysis based on RMSD, RMSF, RG and SASA plots have demonstrated the behavior of these drugs during the entire simulation. Specifically, for *Ts*-PPase our target study, drug alendronate revealed pronounced outcomes with strong molecular interactions at the active pocket key amino acids, that worked together to force alendronate to be stable at the active pocket during the entire period of simulation. Surprisingly, these results were far better than albendazole which conserved only one new H-bond interaction throughout the MD simulation. According to our obtained results, we proposed that alendronate could have anti-parasitic effects and consequently can be a good candidate to treat *T. spiralis* infections based on an extensive in vivo study. The thing that pushes our findings is that, alendronate is currently used to treat bone deficiency, besides its availability, low cost and safety, compared to albendazole’s side effects. Furthermore, ALN may offer additional advantages in the treatment of trichinellosis beyond the standard anthelmintic therapies. These potential benefits include its anti-inflammatory properties, which could help alleviate muscular complications associated with trichinellosis, as well as its inhibitory effects on angiogenesis, which may impact muscle larvae and the formation of nurse cells. However, further validation through in vivo experimental studies including; parasite burden, histopathological analysis (intestinal and muscle tissue sections), immunological and molecular markers assessment (cytokine analysis *i.e.,* IL4, IL10, TNF-*α*, IFN-*γ*), and clinical and biochemical parameters (liver and kidney function tests) are essential to validate the efficacy, pharmacokinetics, and safety of ALN in a physiological environment.

## Supplementary Information

Supplementary file 1.

## Data Availability

The data supporting the findings of this study are available within the article and its supplementary materials.
