# Supplementary material for: Alendronate repositioning as potential anti-parasitic agent targeting Trichinella spiralis inorganic pyrophosphatase, in vitro supported molecular docking and molecular dynamics simulation study
Source: BMC Chem. 2025 May 6;19(1):119. doi: 10.1186/s13065-025-01468-4 (PMC12057173; doi:10.1186/s13065-025-01468-4)
Supplement: Supplementary file 1 [file 13065_2025_1468_MOESM1_ESM.docx]

**Supplementary material**

**Alendronate Repositioning as Potential Anti-Parasitic Agent Targeting *Trichinella spiralis* Inorganic Pyrophosphatase, In vitro Supported Molecular Docking and Molecular Dynamics Simulation Study**

**Marmar A. Hanafy ^a^, Doaa A. Nassar ^a^, Fatima M. Zahran ^a^, Magdy M. D. Mohammed ^b,*^**

*^a^ Department of Parasitology, Faculty of Medicine, Ain Shams University, Cairo, Egypt.*

*^b^ Pharmacognosy Department, Pharmaceutical and Drug Industries Research Institute, National Research Centre, Dokki-12622, Giza, Egypt.*

**Abstract**

Trichinellosis represents great public health and economic problems worldwide. Moreover, the development of parasitic resistance against conventional anthelminthic treatment led to the urgent search for new therapeutic strategies, including drug repurposing. Bisphosphonates have been used to inhibit the growth of many parasites, and have also emerged as promising candidates for the treatment of cryptosporidiosis and amoebic liver abscess. Alendronate is a second-generation bisphosphonate that is widely used for the treatment and prevention of osteoporosis. Till date, there is not enough data on the effect of this drug on Trichinella spiralis and it is unknown whether the regular use of this drug in osteoporotic patients may alter the course of the infection.

In vitro study showed that, a significant lethal effect of alendronate on both adult worms and juveniles, with severe tegumental damage in the form of fissures in the cuticle, widening of the hypodermal gland, and flattening of the cuticular annulation, ending with the appearance of multiple vesicles and large cauliflower masses. In silico molecular docking outcomes unveil the potential inhibition of alendronate against *T. spiralis* surface proteins (*i.e.,* *Ts*-SP, *Ts*-PPase, *Ts*-MAPRC2, *Ts*-TS, *Ts*-MIF, etc.), via its ability to interact with the key amino acids at the active pocket, in addition, the molecular dynamic simulation of *Ts*-PPase_alendronate complex proved its stability during simulation period for 100 ns, the thing that highlights its potential as a valuable drug candidate for the treatment of trichinellosis and warrant its further investigation in animal models.

**Keywords:** Alendronate, Albendazole, *Trichinella spiralis*, Scanning electron microscopy,

Molecular docking, Molecular dynamic simulation.

**S-1:** 2D interactions of ABZ and ALN with *Ts*-SP

**S-2:** 2D interactions of ABZ and ALN with *Ts*-MAPRC2

**S-3:** 2D interactions of ABZ and ALN with *Ts*-5by6

**S-4:** 2D interactions of ABZ and ALN with *Ts*-1hfo

**S-5:** 2D interactions of ABZ and ALN with *Ts*-CF1

**S-6:** 2D interactions of ABZ and ALN with 8xvf


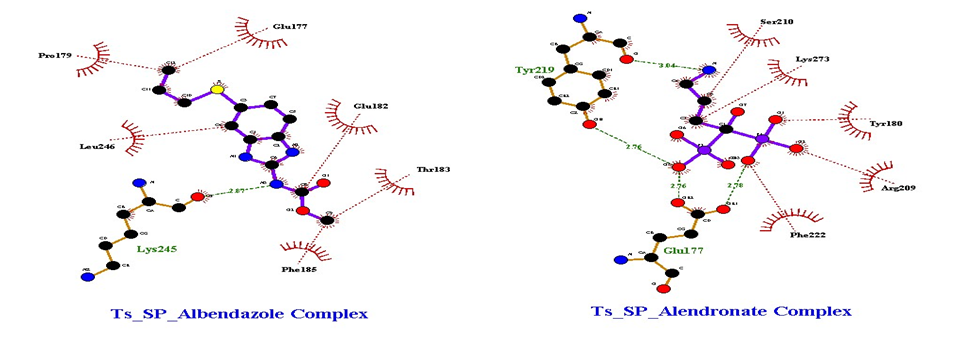


**S-1**


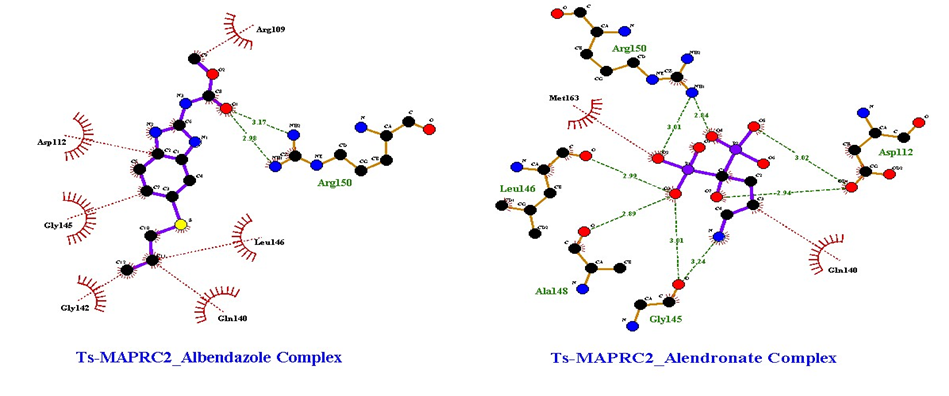


**S-2**


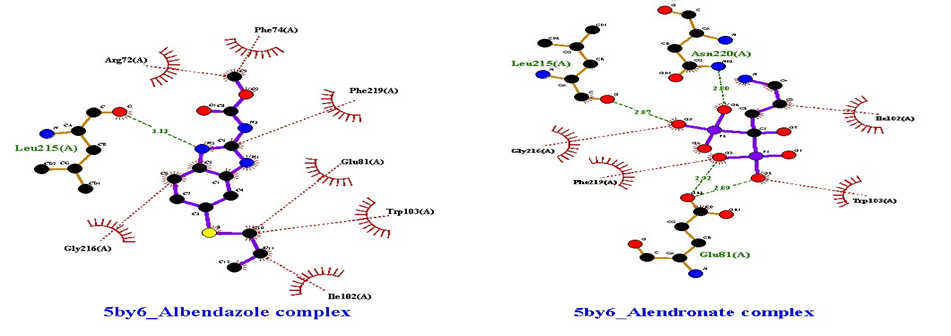


**S-3**


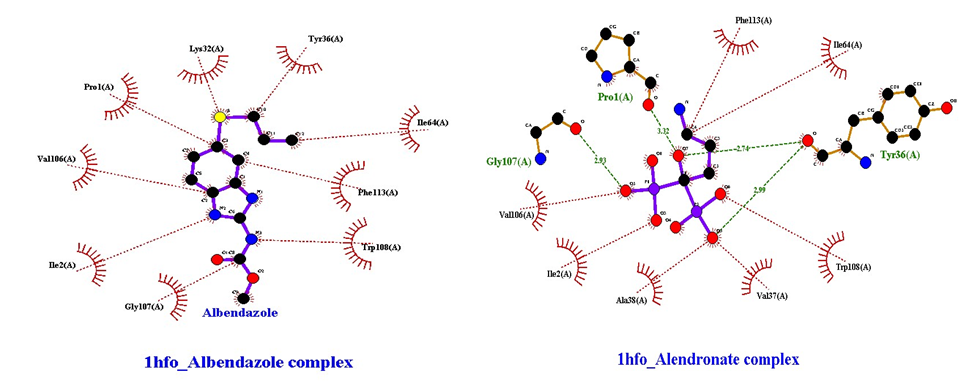


**S-4**


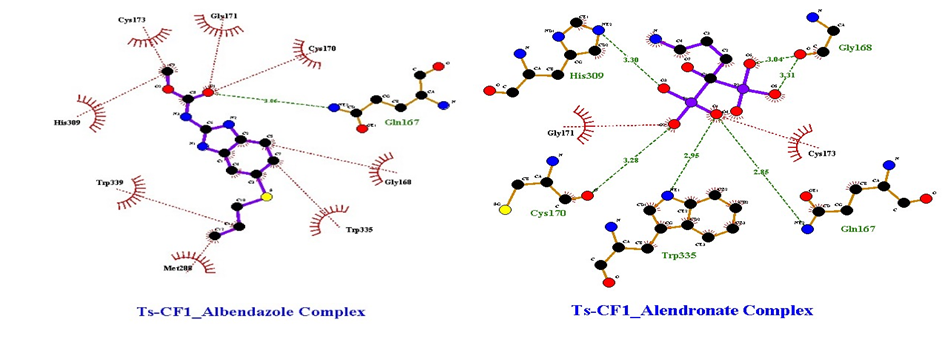


**S-5**


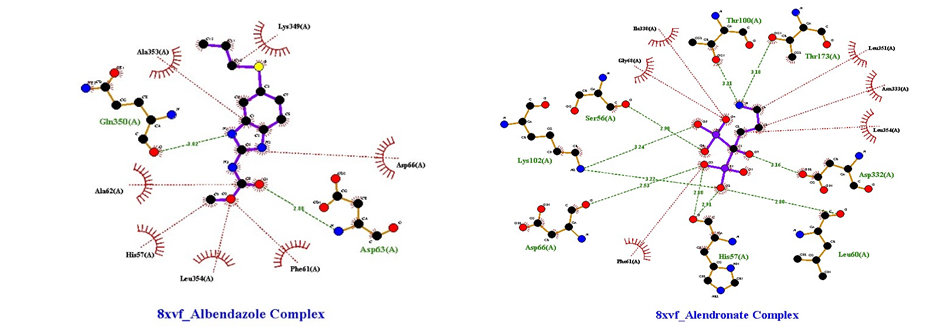


**S-6**

**S-7. The H-bonds donor and acceptor and their occupancy**

| **Ligands** | **HB-Donor** | **HB-Acceptor** | **Occupancy** |
| --- | --- | --- | --- |
| **ABZ** | LIG1-Side | ASP235-Side | 0.26% |
|  | LIG1-Side | TYR176-Side | 0.27% |
|  | LIG1-Side | ASP203-Side | 11.14% |
|  | LIG1-Side | ARG161-Side | 0.01% |
|  | LIG1-Side | THR279-Side | 5.30% |
|  | TYR275-Side | LIG1-Side | 0.63% |
|  | TRP271-Side | LIG1-Side | 0.15% |
|  | TYR172-Side | LIG1-Side | 0.05% |
|  | LYS139-Side | LIG1-Side | 1.26% |
|  | ARG161-Side | LIG1-Side | 0.31% |
|  | LYS276-Side | LIG1-Side | 0.01% |
|  | LYS156-Side | LIG1-Side | 0.03% |
|  | THR279-Side | LIG1-Side | 0.04% |
| **Total occupancy** |  |  | **19.46%** |
| **ALN** | LIG1-Side | ASP235-Side | 5.11% |
|  | LIG1-Side | ASP203-Side | 14.38% |
|  | LIG1-Side | ASP200-Side | 44.70% |
|  | LIG1-Side | TYR176-Side | 0.26% |
|  | LIG1-Main | PRO201-Main | 0.04% |
|  | LIG1-Side | TYR275-Side | 1.45% |
|  | LIG1-Main | ASP203-Side | 0.01% |
|  | LIG1-Side | PRO201-Main | 1.11% |
|  | LIG1-Main | GLU141-Side | 0.01% |
|  | LIG1-Main | TYR176-Side | 0.01% |
|  | LIG1-Main | TYR275-Side | 0.07% |
|  | LIG1-Side | ASP198-Side | 5.71% |
|  | LIG1-Main | THR234-Main | 0.06% |
|  | LIG1-Main | GLU233-Side | 0.41% |
|  | LIG1-Main | ASP235-Side | 0.14% |
|  | LIG1-Main | ASP198-Side | 0.01% |
|  | LIG1-Main | THR279-Side | 0.01% |
|  | LYS237-Side | LIG1-Side | 0.85% |
|  | LYS139-Side | LIG1-Side | 1.42% |
|  | TYR275-Side | LIG1-Side | 0.41% |
|  | LYS139-Side | LIG1-Main | 3.38% |
|  | TYR275-Side | LIG1-Main | 1.02% |
|  | TYR172-Side | LIG1-Main | 0.36% |
|  | LYS276-Side | LIG1-Main | 5.57% |
|  | THR279-Side | LIG1-Main | 0.01% |
| **Total occupancy** |  |  | **86.51%** |
